# Sperm RNA landscape during sexual maturation in Duroc boars

**DOI:** 10.1186/s12864-025-12490-0

**Published:** 2026-01-21

**Authors:** Asmita Shrestha, Maren van Son, Adnan Hashim, Soudabeh Rouzbehani, Gregor D. Gilfillan, Urszula Berge, Elisabeth Kommisrud, Anne Hege Alm-Kristiansen

**Affiliations:** 1 CRESCO, Centre for Embryology and Healthy Development, University of Inland Norway, Hamar, Norway; 2 Norsvin SA, Hamar, Norway; 3 CRESCO, Centre for Embryology and Healthy Development, University of Oslo, Oslo, Norway; 4 Department of Medical Genetics, Oslo University Hospital, Oslo, Norway

**Keywords:** miRNA, Porcine spermatozoa, Puberty, Sexual maturation

## Abstract

**Background:**

Sexual maturation in boars impacts reproductive efficiency in swine production, yet the molecular mechanisms underlying this developmental transition remain poorly understood. This study aimed to investigate the transcriptomic changes in sperm from Duroc boars during sexual maturation, conducting a longitudinal analysis. The total RNA and miRNA profiles from the same individuals (*n* = 6) at puberty (7.24 ± 0.39 months) and sexual maturity (10 ± 0.40 months) were compared, identifying molecular signatures associated with reproductive development. Total RNA sequencing (Illumina NovaSeq-6000) and miRNA sequencing (Illumina NextSeq-500) were performed on all 12 paired samples (6 boars at 2 time points), followed by differential expression analysis using a paired statistical model in DESeq2 to account for repeated measures.

**Results:**

Differential expression analysis identified 60 differentially expressed genes using stringent criteria (adj *P* < 0.05, |log_2_FC| ≥ 0.5), with 65% upregulated in sperm of boars 10-months versus 7-months of age. Key upregulated genes included *NCLN*,* RGS12*, *CIB2*, *FOXP4*, *PHC1*, *CDC25B* and *AKAP1*, while *HSP90AA1*,* EVI5*,* FSIP2*,* VDAC3*, and *ALMS1* were key downregulated genes. Furthermore, Gene ontology analysis revealed significant enrichment of 11 biological processes, mostly related to reproductive development, and four molecular functions (transferase activity, transferring phosphorus-containing groups, protein serine/threonine kinase activity, protein kinase activity and signal sequence binding). Additionally, our miRNA analysis identified six differentially abundant miRNAs (adj *P* < 0.05 & |log_2_FC| ≥ 0.5); ssc-miR-193a-5p, ssc-miR-574-3p, ssc-miR-126-3p, ssc-miR-196a, ssc-miR-210 were upregulated, and ssc-miR-338 showed downregulation in 10-months age. Integrated analysis of differentially expressed mRNA and predicted miRNA targets identified 18 miRNA-mRNA regulatory pairs, enriched in pathways related to cell cycle processes and chromatin binding, suggesting coordinated regulation across RNA biotypes in sperm during sexual maturation.

**Conclusions:**

Our study reveals coordinated transcriptomic shifts in boar sperm during sexual maturation. Most genes show increased RNA abundance at 10 months of age, with enrichment in terms of reproductive development. Several miRNAs appear to regulate these changes through targeted mRNA interactions. These findings expand our understanding of the biological processes underlying sexual maturation in pigs.

**Supplementary Information:**

The online version contains supplementary material available at 10.1186/s12864-025-12490-0.

## Introduction

Sexual reproduction in mammalian males depends on producing functionally competent spermatozoa through spermatogenesis and their subsequent maturation [1]. Spermatogenesis begins in the testes, where haploid sperm develop morphologically but remain functionally immature. These developing sperm only acquire progressive motility and fertilization capacity during their transit through the epididymis from the testis [1]. Within the epididymis, sperm undergo biochemical and physiological transformations, including membrane remodeling, protein modifications and the development of motility and fertilization potential [2]. This maturation process is essential for producing sperm capable of navigating within the female reproductive tract to reach, penetrate and fertilize an egg [1].

In boars, sperm production begins at puberty (around 5–6 months of age), while sexual maturity and optimal semen functionality develop gradually from 8 to 9 months and proceed until approximately 24 months, though individual and breed variations exist [3–5]. The progression toward sexual maturity is reflected in several measurable parameters, including sperm motility, total sperm number, ejaculate volume, sperm concentration and morphology [6, 7]. Duroc boars show an increase in ejaculate volume, sperm concentration, total sperm count, motility, normal morphology, and sperm count with normal acrosome as they mature from 230 days to 270 days, while the percentage of morphologically defective sperm decreases during this period [7]. Similar improvements continue in most pig breeds (Polish Large White, Polish Landrace, Pietrain, Hamshire and Duroc), with a progressive rise in ejaculate volume and sperm concentration from 9 months onward [5]. These parameters are important as they determine the total sperm output, which dictates the number of artificial insemination (AI) doses that can be produced from a single ejaculate (e.g., cervical AI typically targets ~ 2–3 × 10^9^ sperm/dose), thereby maximizing a boar’s economic efficiency and the dissemination of superior genetics [8]. Histological examination provides further support of this developmental timeline. In Duroc testes, spermatozoa and seminiferous tubule lumina become detectable at 150 days, with a large number of spermatocytes visible at 270 days, while these structures are absent at earlier stages (75 and 130 days) [3, 4]. Seminiferous tubule diameter also increases between 75 and 270 days, reflecting continuous testicular maturation [3].

Alongside these age-related macroscopic improvements in sperm quality parameters, there is an increasing interest in the underlying molecular changes during the transition from puberty to sexual maturity. Transcriptomic analysis offers valuable insights into these molecular-level changes in sperm. While spermatozoa were traditionally considered merely carriers of paternal genetic material and believed to be transcriptionally silent due to minimal cytoplasm and absence of intact ribosomal RNAs (rRNAs), it has been reported the presence of rich RNA landscape within spermatozoa [9, 10]. Boar sperm contains diverse RNA populations, including messenger RNAs (mRNAs), micro RNAs (miRNAs), short noncoding RNAs (sncRNAs), small nucleolar RNAs (snoRNAs), small nuclear RNAs (snRNAs), Piwi interacting RNAs (piRNAs), transfer RNAs (tRNAs), ribosomal RNAs (rRNAs), mitochondrial ribosomal RNAs (MtrRNAs), and mitochondrial transfer RNAs (MttRNAs) [11–13]. Several of these sperm RNAs are reported to be associated with sperm maturation, functionality and fertility outcomes in boars [14].

Early characterization of porcine sperm RNA by Yang and colleagues in 2009 [12], using expressed sequence tags (ESTs), revealed a fragmented RNA profile containing transcripts encoding proteins involved in spermatogenesis such as protamine 1 (*PRM1*) and protamine 2 (*PRM2*), as well as early embryonic development, including sperm-specific antigen 2 (*SSFA2*) and sestrin 1 (*SESN1*). This pioneering work was expanded through RNA sequencing (RNA-Seq) approaches, which identified 4436 genes expressed in boar sperm that reflect seasonal changes, including highly abundant transcripts of mitochondrial origin and key spermatogenesis regulators like *PRM1* and ornithine decarboxylase antizyme 3 (*OAZ3*) [15, 16].

RNA-Seq analysis has further identified many genes functionally relevant to sperm motility [17], morphology [11], fertilization capacity [18], and freezability [19, 20]. For instance, comparison between fresh and frozen-thawed boar sperm revealed 567 mRNAs and 135 miRNAs to be differentially expressed [19]. Similarly, Alvarez-Rodriguez et al. [18] observed 347 up-regulated and 174 down-regulated RNA transcripts in high-fertility boars versus low-fertility boars, including transcripts encoding proteins involved in sperm motility, such as cation channel sperm associated auxiliary subunit gamma (*CATSPERG*).

Non-coding RNAs, especially miRNAs, have attracted considerable research interest due to their involvement in regulating boar sperm quality and function. Curry et al. [21] reported differential expression of miRNAs (including miR-22, let-7a, let-7d, and let-7e) in sperm with abnormal morphology or reduced motility. Chang et al. [22] identified 15 differentially expressed miRNAs between fresh ejaculated and cauda epididymal spermatozoa in Landrace boars, with eight being downregulated (including ssc-let-7 family members, ssc-miR-26a, ssc-miR-98) and seven upregulated (including ssc-miR-19b, ssc-miR-34c). These miRNAs target genes involved in key signaling pathways such as cell-cycle regulation, and apoptosis [22].

Martinez et al. [23] found four differentially expressed miRNAs (miR-1285, miR-92a, miR-34c, and miR-30) between ejaculate and cauda epididymal spermatozoa, while also observing varying abundance of miR-182, miR-1285, miR-191, and miR-96 between high and low fertility boars, suggesting these miRNAs are involved in fertility. In a comprehensive profiling study, Luo et al. [24] revealed distinct miRNA expression patterns across reproductive tissues, with approximately 15% of miRNAs co-expressed in Landrace testis, epididymis and sperm, mirroring the physiological relationship between these tissues. Particularly, miR-34 showed remarkably higher abundance in sperm, compared to the other reproductive tissues [24].

Gòdia et al. [11] have revealed correlations between RNA abundance and 25 sperm quality parameters in Pietrian boars, demonstrating the complex molecular basis of sperm function and fertility. This examination identified highly abundant mRNAs (*PRM1*, *OAZ3*, DnaJ heat shock protein family member B8 (*DNAJB8)*, tripeptidyl peptidase 2 (*TPP2*), transition protein 1 (*TNP1*)) and miRNAs (ssc-miR-30d, ssc-miR-34c, ssc-miR-30c-5p, ssc-miR-191, members of the let-7 family, and ssc-miR-425-5p) in boar sperm associated with sperm biology [11].

Despite considerable progress in understanding sperm biology through transcriptomics, there remain significant gaps in our knowledge concerning the transcriptomic changes that characterize the transition from puberty to sexual maturity in boars. Previous studies have primarily examined RNA level in boar sperm in relation to season, anatomy, sperm quality, fertility, and cryopreservation using a cross-sectional design comparing different individuals [11, 16, 18, 20]. Limited understanding exists about which genes become systematically enriched or depleted as boars mature sexually, and whether these molecular shifts actively contribute to enhanced sperm performance or are simply developmental byproducts. Understanding these developmental changes at a molecular level has gained interest as breeding companies are increasingly motivated to introduce younger boars earlier in breeding programs based on genomic selection [25].

Duroc is widely used as a terminal sire in commercial pork production systems and is valued for its superior robustness, growth, and meat quality. The breed was founded by crossing Red Durocs (New York) and Jersey Reds (New Jersey) lines [26]. In many commercial programs, market pigs are produced by mating Duroc boars to Yorkshire × Landrace dam lines [26]. In Norway, Duroc is the main sire line with nucleus testing of around 5,000 young boars each year [27]. Given that the reproductive performance of these sires is a critical economic factor as it directly affects AI dose output and fertility, gaining knowledge on their sperm maturation provides vital insights for optimizing breeding strategies in these commercial lines.

This study aimed to perform comparative transcriptomic profiling of sperm collected from the same Duroc boars at both puberty (7.24 ± 0.39 months) and sexual maturity (10 ± 0.40 months) using a longitudinal study design. Our primary objectives were to: (i) identify differences in abundance of RNAs and miRNAs in sperm between these two developmental time points, and (ii) explore the biological pathways and molecular mechanisms associated with sexual maturation. We hypothesized that sperm from sexually mature boars would exhibit distinct transcriptomic signatures compared to those from the same boars at puberty, reflecting molecular adaptations that enhance reproductive function. These insights would provide a unique window into developmental changes in boar sperm at the molecular level, which might be utilized to optimize selection criteria in breeding programs.

## Materials and methods

### Experimental design

This study examined 6 *Sus scrofa* boars of the Duroc breed over a three-month time span from March to June 2023. The boars were sourced from a commercial breeding line used by Norsvin SA, a pig breeding company in Norway. To track transcriptomic transitions linked to sexual maturity, semen was collected from the same individuals at two time points, at 7.24 ± 0.39 months (31.48 ± 1.71 weeks) (pubertal) and 10 ± 0.40 months (43.48 ± 1.74 weeks) (mature) of age.

At the start of the study, the pubertal boars were placed in individual 4 m² pens within an isolation unit, adhering to guidelines set by the Norwegian Food Safety Authority for animals of this size. This phase served as a quarantine period and an opportunity to train boars for semen collection procedures. Semen samples were collected once the animals showed interest in mounting and were physically capable of producing semen. The boars were transferred to an Artificial Insemination (AI) center at around 8 months old (~ 37 weeks), where they were housed in larger 6 m² pens. Semen samples (sperm-rich fraction of ejaculate) were obtained using the gloved-hand technique, always carried out by the same trained personnel to ensure consistency. The collection procedure was entirely non-invasive; no anesthesia or euthanasia was required at any stage of the study. Ejaculate was initially collected in new disposable paper cups (normal procedure of Norsvin SA) and immediately transferred with sterile pipette tips into sterile 50 mL polypropylene tubes. For collection, the boars were briefly moved from their home pens to a separate area. Environmental conditions, including ventilation, lighting, and temperature, were kept consistent across both the isolation unit and the AI center to avoid introducing factors that might affect metabolic or physiological measurements. All boars were provided with the same balanced commercial diet throughout the study.

### Sample pre-processing

Immediately after collection at the AI station at Norsvin SA, sperm concentration was determined at the AI station using the NucleoCounter^®^ SP-100™ (Chemometec, Allerød, Denmark). Samples were then transported to the INN laboratory in a Styrofoam box within 30 min, and processing of the sample began immediately upon arrival. For semen quality assessment, samples were diluted in Androstar^®^ Plus extender (Minitube, Germany) to 25 × 10^6^/mL and incubated at 38 °C for 20 min. Motility was then evaluated using the Sperm Class Analyzer (Microptic SL, Spain). Spermatozoa were identified based on a head area of 20–80 μm², with the motility threshold set at an average path velocity greater than 10 μm/s. The specific quality parameters for all 12 samples used in this study are detailed in Table 1. This study cohort represents boars that were successfully retained in the stud for routine collections, with all samples exhibiting above 80% of motility.

**Table 1:** Semen quality parameters for 12 samples collected from six Duroc boars at 7.24 ± 0.39 and 10.00 ± 0.40 months of age. Values shown are sperm motility (%) evaluated in the extended semen and concentration (10^6^/mL) measured in raw semen

Boar	~ 7 months		~ 10 months	
	Motility (%)	Conc. (10^6^/mL)	Motility (%)	Conc. (10^6^/mL)
B8	92.76	679.70	92.98	416.8
B9	85.15	156.7	90.88	107.1
B11	91.04	507.50	94.09	291.2
B13	81.38	586.3	80.69	651.4
B14	85.02	162.0	87.65	246.2
B15	91.10	379.40	89.90	287.6
Mean ± SD	87.74 ± 4.51	522.20 ± 150.69	89.36 ± 4.82	333.38 ± 184.81

A fresh semen sample containing 200 million sperm per ml was centrifuged at 600×g for 10 min at room temperature (RT) to separate sperm from the seminal plasma. After discarding the supernatant (seminal plasma), the sperm pellets were washed with 1 mL phosphate-buffered saline (PBS) (centrifuged at 600×g for 10 min at RT). Next, the somatic cell lysis was performed in the sample as per Goodrich et al. [28] with slight modification. The samples were kept on ice for 30 min with 1 mL of somatic cell lysis buffer (0.1% SDS, 0.5% Triton X-100 in nuclease-free water) and thereafter washed by centrifuging at 200 × g for 15 min at 4 °C. The samples were thereafter examined using phase-contrast microscopy to ensure the absence of somatic cells. Following this examination, the sperm were washed twice with 1 mL PBS, each time centrifuged at 600×g for 10 min at 4 °C. Finally, the sperm pellets were snap-frozen and stored in liquid nitrogen until further analysis.

### Total RNA isolation and quality assessment

Frozen spermatozoa were thawed in hands and immediately processed for lysis. To lyse the sperm cells, 1 ml of Qiazol (Qiagen, Germany) and 120 µL of tris(2-carboxyethyl)phosphine (TCEP) (Sigma-Aldrich, Saint-Louis, MO, USA) were added to the sperm pellet. The mixture was vortexed for 5 min, incubated at 56 °C for 60 min and then incubated at RT for another 5 min. To confirm successful lysis of the spermatozoa, phase-contrast microscopy was used. For RNA isolation, the manufacturer’s kit protocol (miRNeasy Mini Kit, Qiagen, Germany) was followed with slight modifications. To each sample lysate, 200 µL of chloroform was added before centrifugation at 12,000×g at 4 °C for 15 min. The upper aqueous phase was transferred to a new collection tube. 100% ethanol (1.5 times recovered aqueous phase) was added to the aqueous phase and mixed thoroughly. The sample was then loaded into the RNeasy Mini spin column and centrifuged at 8000×g for 20 s at RT. Washing was performed with Buffer RWT (prepared with isopropanol) centrifuged at 8000×g for 20 s at RT. On-column DNase digestion was performed by adding 10 µL DNase I stock solution to 70 µL Buffer RDD as instructed in the kit RNase-Free DNase Set (Qiagen, Germany). Washing of the column was performed subsequently by 350 µL Buffer RWT, and 500 µL Buffer RWT with centrifugation at 8000×g for 20 s at RT. Additional washing of the spin column by 500 µL Buffer RPE was done by centrifuging at 8000×g for 2 min to dry the spin column membrane at RT. To eliminate any possible carryover of Buffer RPE or residual flow-through, the spin column was placed into a new 2 mL collection tube and centrifuged at full speed for 1 min. Finally, total RNA elution was done with 40 µL of RNase-free water. The quantity of RNA was measured using Qubit™ 4 fluorometer with Qubit™ High Sensitivity (HS) kit (Thermo Fisher Scientific, USA). Assessment of RNA quality was performed using 4150 TapeStation system and TapeStation Analysis Software (4.1.1) with High Sensitivity RNA ScreenTape (Agilent Technologies, Santa Clara, CA, USA) following the manufacturer’s protocol. Lack of somatic cell contamination was confirmed by the non-detection of 18 S and 28 S rRNA peaks in the TapeStation analysis [12, 29]. Similarly, absence of DNA contamination was confirmed using *PRM1* TaqMan™ Gene Expression Assay (Applied Biosystems, CA, USA, Assay ID: Ss03383652_u1) by comparing RT-qPCR reactions with and without reverse transcriptase (-RT controls) following the protocol described in [30].

### Total RNA sequencing

Using 4 µl total RNA (8.8–61.2 ng), rRNA was blocked from cDNA extension using the QiaSeq FastSelect HMR reagents (Qiagen, Germany), following the “-rRNA and/or -Globin with the NEBNext Ultra II Directional Library Prep Kit” procedure in the manufacturer´s handbook v12/21, including the “partially degraded RNA (RIN 2–6)” variations. Sequencing libraries were thereafter prepared with the Ultra II Directional Library Prep Kit (NEB) according to manufacturer´s instructions version 5.0_12/22 from step 4.2 onwards, with dilution of the NEB adapter 1:25 and indexed adapters. Libraries were amplified for 16 PCR cycles. Sequencing was performed on a NovaSeq-6000 instrument (Illumina) on an SP flowcell running RTA v3.4.4 with 1% PhiX added, with 150 bp paired end sequencing. Demultiplexing into individual sample FASTQ datasets was performed with bcl2fastq v2.20.0.422.

### miRNA sequencing

miRNA libraries were prepared using the QiaSeq miRNA library kit (Qiagen, Germany). Inputs ranged from 5.5 to 38.3 ng RNA. Adapters were diluted accordingly, following the manufacturer´s recommendations, and samples were amplified with 18 cycles of PCR. Samples were indexed with unique adapters to allow pooling on a single sequencing run. Sequencing was performed on a NextSeq-500 instrument (Illumina) running RTA v2.11.3 with 1% PhiX added, with 82 cycles of sequencing. Demultiplexing into individual sample FASTQ datasets was performed with bcl2fastq v2.20.0.422.

### Total RNA-Seq and miRNA-Seq analysis

Raw fastq files of both total RNA-Seq and miRNA-Seq were subjected to quality control using FastQC (v0.12.1) [31]. For total RNA-seq, adapter sequences and low-quality bases were removed using Trim Galore (v0.6.10) [32] with the following parameters: quality cutoff of 20, stringency of 3, and a minimum read length of 35 bases. The trimming was executed in paired-end mode, retaining unpaired reads and trimming ambiguous “N” bases. Post-trimming quality was again confirmed by running FastQC on the trimmed sequences. The *Sus scrofa* (Sscrofa 11.1) reference genome and its corresponding GTF annotation were obtained from Ensembl release 112 [33]. The reference genome was indexed for alignment using HISAT2’s (v2.2.1) hisat2-build [34]. Trimmed paired-end reads were aligned to the *Sus scrofa* genome using HISAT2 (v2.2.1) [34]. Alignment output (SAM files) was converted to BAM files using SAMtools (v1.16.1), then sorted and indexed. To account for PCR duplicates, duplicate marking was performed using Picard (v3.0.0) [35]. The duplicate-marked BAM files were indexed, and post-marking alignment statistics were obtained. Gene-level counts matrix were generated using featureCounts (Subread v2.0.4) [36] with the *Sus scrofa* ensembl GTF annotation file [33]. Parameters for featureCounts included paired-end mode, counting reads mapping to exons (using -t exon and -g gene_id), and a strandedness option (-s 2).

For miRNA-seq, the standalone version of sRNAbench from the sRNAtoolbox suite [37] was used to identify both known *Sus scrofa* miRNAs (based on miRbase v21) and novel miRNA candidates from the small RNA sequencing data. This analysis was performed following the default parameters provided by the software. The “mature_sense.grouped” output file from sRNAbench, which contained the raw expression values of the miRNAs, was used to create the count matrix. This matrix was then used as input for DESeq2 [38] to perform the differential expression analysis. Only known miRNAs were retained for downstream analyses, and novel miRNA candidates were not further considered.

### Differential RNA abundance analysis of total RNA-Seq and miRNA-Seq data

The DESeq2 package (v 1.46.0) from Bioconductor [38] was used to perform differential RNA abundance analysis for both total RNA-Seq and small RNA-Seq in R [39]. For both datasets, a paired design (‘~ Individual + Age’) was implemented to account for individual variation while focusing on changes between Pubertal and Mature boar groups. To ensure robust analysis, we filtered both datasets to include only genes with a minimum of 10 read counts in at least 6 samples following recommended practice [40]. Differentially expressed genes (DEGs) were identified using the following criteria: adjusted *P*-values < 0.05 (Benjamini-Hochberg correction) and absolute log_2_ fold change ≥ 0.5. In this paper, DEGs refers to differential abundance of sperm RNA. For total RNA-Seq data, differentially expressed Ensembl gene IDs were converted to gene names using biomaRt (v 2.62.0), by querying the Ensembl BioMart database (‘sscrofa_gene_ensembl’) to retrieve external gene names, Entrez IDs, gene descriptions, and biotypes [41].

For miRNA analysis, target gene prediction was performed using the multiMiR package (v 2.4.0) [42]. Due to the lack of Sus scrofa support in multiMiR and limited miRNA-target annotation in pigs, differentially expressed porcine miRNAs meeting the criteria (adj *P* < 0.05 and |log_2_FC| ≥ 0.5) were manually mapped to human orthologs based on sequence conservation. Target prediction was conducted using the get_multimir() function with the following parameters: org = “hsa”, table = “all” to retrieve miRNA target interactions from multiple integrated databases including eight predicted databases (DIANA-microT, ElMMo, MicroCosm, miRanda, miRDB, PicTar, PITA, and TargetScan) and three validated databases (miRecords, miRTarBase, and TarBase) [42]. The resulting human gene targets were then mapped back to porcine orthologs using the gorth() function from gprofiler2 (v 0.2.3) [43] by querying the pig genome database (‘sscrofa_gene_ensembl’).

Gene ontology (GO) enrichment analysis for both datasets was performed using clusterProfiler (v 4.14.4) [44] in conjunction with the *Sus scrofa* OrgDb (org.Ss.eg.db) (v 3.20.0) [45, 46]. Differentially expressed genes from total RNA-Seq and predicted target genes from miRNA-Seq were tested for enrichment in biological processes, molecular functions, and cellular components. GO enrichment analysis was performed using Benjamini-Hochberg correction, with significantly enriched terms identified at q-value < 0.05.

For integrative analysis to identify miRNA-mRNA regulatory pairs, predicted miRNA targets were intersected with differentially expressed mRNAs from our dataset (adj *P* < 0.05 and |log_2_FC| ≥ 0.5), showing inverse and positive expression patterns. Specifically, predicted mRNA targets of downregulated and upregulated miRNAs were matched with downregulated and upregulated miRNAs. The resulting miRNA-mRNA regulatory pairs were annotated with gene symbols using Ensembl BioMart [41].

## Results

### Sequencing metrics

For total RNA-Seq, 12 sperm libraries generated 60.8 ± 9.7 million raw reads per sample (30.41 ± 4.83 million reads per end) (9,183.38 ± 1,459.05 Mbp), with 151 bp paired-end reads. Trimming retained 95.73% ± 4.45% of reads, resulting in 58.18 ± 9.74 million trimmed reads per sample with an average length of 126.20 ± 2.78 bp. Alignment to the *Sus Scrofa* genome achieved overall alignment rate of 78.08% ± 3.06%. We observed Picard duplicate rate of 87.92% ± 3.77%. We obtained 45.48 ± 8.42 million mapped reads per sample, providing sufficient depth for differential expression analysis (Additional File: Table S1).

### RNA landscape and differential expression analysis

Initial characterization of total RNA-Seq data revealed a diverse transcriptional landscape in boar sperm. From the 15,637 genes of all biotypes detected after quality filtering (≥ 10 read counts in at least 6 samples), protein-coding genes constituted the majority (86.91%), followed by long non-coding RNAs (11.56%), with smaller proportions of small nucleolar RNA (0.61%), pseudogenes (0.33%), and other RNA classes (Additional file: Table S2). In our boar sperm transcriptome dataset, the top five abundant protein-coding genes were *PRM1*, SPEM family member 2 (*SPEM2*), *OAZ3*, heat shock protein family B (small) member 9 (*HSPB9*), and ankyrin repeat domain 35 (*ANKRD35*).

**Fig. 1:**
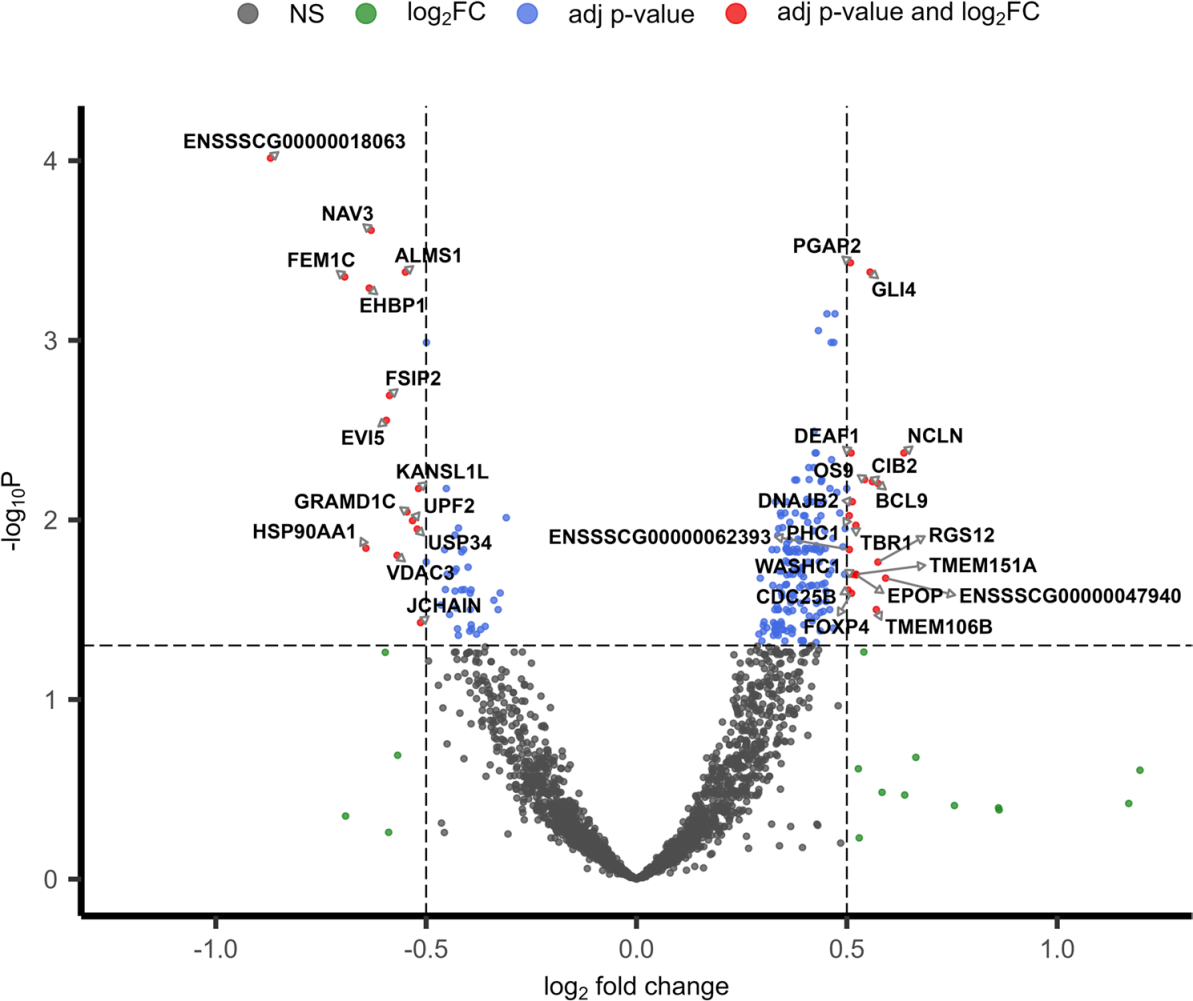
Volcano plot of differential gene expression between sperm samples collected from six Duroc boars (*n* = 12 samples total) at 10 months and 7 months of age. log_2_ fold change (x-axis) is plotted against statistical significance (-log_10_ adj *P*-value, y-axis) for expressed genes. Red dots represent significantly differentially expressed genes meeting both significance (adj *P* < 0.05) and fold change (|log_2_FC| ≥ 0.5) criteria. Blue dots indicate genes with significant *P*-values (adj *P* < 0.05) but below the fold change threshold (|log_2_FC| < 0.5). Green dots show genes with fold change above threshold (|log_2_FC| ≥ 0.5) but lacking statistical significance (adj *P* > 0.05). Grey dots represent genes which are non-significant (NS)

Differential expression analysis between sperm samples from boars at 10-months and 7-months of age initially identified 230 genes with significant *P*-values (adj *P* < 0.05) (Additional file: Table S3). The majority of DEGs were protein-coding genes (221, 96.08%) with smaller contributions from five lncRNAs (2.17%), three pseudogenes (1.30%), and one mitochondrial rRNA (0.43%). Applying additional biological significance criteria (|log_2_FC ≥ 0.5|) refined this to 60 DEGs. These included 39 upregulated genes (65%) and 21 downregulated genes (35%) in mature boars (Fig. 1).

**Fig. 2:**
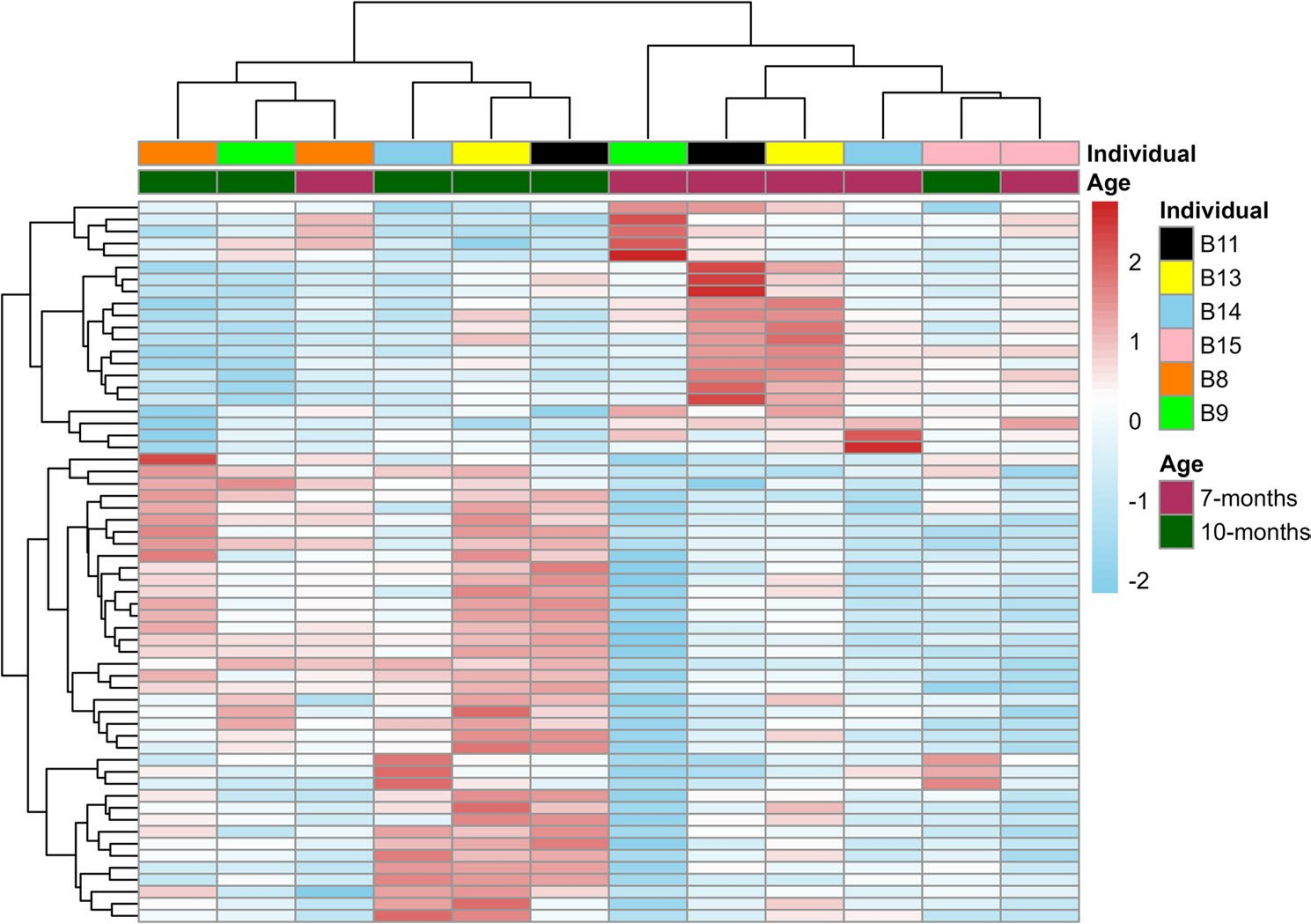
Hierarchical clustering of differentially expressed genes in sperm samples collected from six Duroc boars (*n* = 12 samples total) at 7 months and 10 months of age. Heatmap shows expression patterns of all 60 differentially expressed genes (adj *P* < 0.05 & |log_2_FC| ≥ 0.5) across sperm samples from six Duroc boars at two ages. Each column represents an individual sample with color bars at the top indicating the boar’s identity and age (7-months in maroon, 10-months in dark green). Gene expression values were variance-stabilized and row-scaled, with red indicating higher expression and blue indicating lower expression relative to the mean across samples

Hierarchical clustering of all the DEGs showed an age-dependent expression profile, with samples clustering by age rather than by individual boar, with two exceptions (Fig. 2).

### Key genes identified in sperm maturation

Analysis revealed a mitochondrial rRNA (ENSSSCG00000018063) as the most significantly altered DEG (Table 2) (log_2_FC = −0.9, adj *P* = 9.67 × 10^− 5^). Other highly significant DEGs included neuron navigator 3 (*NAV3*) (log_2_FC = −0.6), post-GPI attachment to proteins 2 (*PGAP2*) (log_2_FC = 0.5), ALMS1 centrosome and basal body associated protein *(ALMS1*) (log_2_FC = −0.5), and GLI family zinc finger 4 (*GLI4*) (log_2_FC = 0.6), all with adj *P* < 4.17 × 10^− 4^. Several of these top-ranked genes exhibited contrasting expression patterns, with both up- and down-regulated genes represented among the most significant changes (Table 2), highlighting the complex transcriptional remodeling that accompanies sperm maturation.

**Table 2:** Results from DESeq2 expression analysis for the top 10 most significantly differentially expressed genes in sperm samples from six boars at 10-months, compared to 7-months of age (*n* = 12 samples total). Genes are ranked by adjusted *P*-value (statistical significance), showing both up-regulated and down-regulated genes. All genes are protein coding genes except ENSSSCG00000018063 (Mt_rRNA). The table includes ensembl gene ID, external gene name, base mean normalized values (baseMean), log_2_ fold change (log_2_FC), standard error of the fold change (lfcSE), Wald test statistics (stat) and adjusted *P*-value (adj *P*-value). CPM values were calculated as counts per million mapped fragments from DESeq2 size-factor normalized counts and mean ± SD were calculated within each age group

Ensgene ID	External Gene Name	baseMean	log_2_FC	lfcSE	stat	adj *P*	CPM 7 mo (mean ± SD)	CPM 10 mo (mean ± SD)
ENSSSCG00000018063	*Novel gene*	3780.19	−0.9	0.16	−5.48	9.67 × 10^− 5^	265 ± 163.25	141.97 ± 61.23
ENSSSCG00000029249	*NAV3*	5125.87	−0.6	0.12	−5.19	2.44 × 10^− 4^	334.71 ± 71.5	217.84 ± 53.28
ENSSSCG00000035653	*PGAP2*	20008.42	0.5	0.10	5.03	3.70 × 10^− 4^	882.69 ± 209.73	1274.14 ± 381.84
ENSSSCG00000037977	*ALMS1*	10215.12	−0.5	0.11	−4.91	4.17 × 10^− 4^	657 ± 158.63	444.15 ± 87.06
ENSSSCG00000039881	*GLI4*	4372.87	0.6	0.11	4.91	4.17 × 10^− 4^	191.55 ± 31.84	279.82 ± 30.26
ENSSSCG00000014217	*FEM1C* ^*1*^	1686.26	−0.7	0.14	−4.86	4.43 × 10^− 4^	114.45 ± 60.94	67.32 ± 22.77
ENSSSCG00000008372	*EHBP1* ^*2*^	4150.54	−0.6	0.13	−4.80	5.12 × 10^− 4^	279.77 ± 183.83	167.64 ± 68.84
ENSSSCG00000034994	*Novel gene*	23962.23	0.5	0.10	4.69	7.12 × 10^− 4^	1086.44 ± 251.58	1496.59 ± 388.87
ENSSSCG00000037391	*AKAP1* ^*3*^	39884.72	0.5	0.10	4.69	7.12 × 10^− 4^	1801.75 ± 434.33	2497.66 ± 572.53
ENSSSCG00000001689	*ZNF644* ^*4*^	3683.40	−0.5	0.10	−4.54	1.03 × 10^− 3^	232.82 ± 48.80	164.23 ± 30.83

^1^Fem-1 homolog C

^2^EH domain binding protein 1

^3^A-Kinase anchoring protein 1

^4^Zinc finger protein 644

We next examined genes showing the most pronounced upregulation in sperm collected from boars at 10 months compared to the same boars at 7 months of age (Table 3, adj *P* < 0.05 & |log_2_FC| ≥ 0.5). *Nicalin* (*NCLN*) was the most upregulated gene, followed by regulator of G protein signaling 12 (*RGS12*), *BCL9* transcription coactivator (*BCL9*), transmembrane protein 106B (*TMEM106B*), calcium and integrin binding family member 2 (*CIB2*), *GLI4*, OS9 endoplasmic reticulum lectin (*OS9*), elongin BC and polycomb repressive complex 2 associated protein (*EPOP*), and transmembrane protein 1*51 A* (*TMEM151A*) with log_2_FC values of 0.5 to 0.6. Other important upregulated genes in mature boars identified were polyhomeotic homolog 1 (*PHC1*), cell division cycle 25B (*CDC25B*), and A-kinase anchoring protein 1 (*AKAP1*) (Additional file: Table S4, adj *P* < 0.05, log_2_FC = 0.5).

The most downregulated genes (Table 4, adj *P* < 0.05 & |log_2_FC| ≥ 0.5) revealed a different functional profile. An uncharacterized mitochondrial rRNA showed the highest downregulation followed by *FEM1C*, heat shock protein 90 alpha family class A member 1 (*HSP90AA1*), *EHBP1*, *NAV3*, ectropic viral integration site 5 (*EVI5)*, fibrous sheath interacting protein 2 (*FSIP2*), voltage dependent anion channel 3 (*VDAC3)*, *ALMS1*, GRAM domain containing 1 C (*GRAMD1C*), and UPF2 regulator of nonsense mediated MRNA decay (*UPF2*). Additional file: Table S4 provides comprehensive expression data for all DEGs.

**Table 3:** Results from DESeq2 expression analysis for the top 15 upregulated genes in sperm samples from six boars at 10-months, compared to 7-months of age (*n* = 12 samples total). Genes are ranked by magnitude of log_2_ fold change (log_2_FC ≥ 0.5). All genes are protein-coding. The table includes ensembl gene ID, external gene name, base mean normalized values (baseMean), log_2_ fold change (log_2_FC), standard error of the fold change (lfcSE), Wald test statistics (stat) and adjusted *P*-value (adj *P*-value). CPM values were calculated as counts per million mapped fragments from DESeq2 size-factor normalized counts and mean ± SD were calculated within each age group

Ensgene ID	External Gene Name	baseMean	log_2_FC	lfcSE	stat	adj *P*	CPM 7 mo (mean ± SD)	CPM 10 mo (mean ± SD)
ENSSSCG00000013475	*NCLN*	2068.17	0.6	0.15	4.13	4.23 × 10^− 3^	87.36 ± 21.32	135.58 ± 33.96
ENSSSCG00000047940		2466.01	0.6	0.18	3.23	2.11 × 10^− 2^	105.50 ± 30.03	160.33 ± 56.12
ENSSSCG00000008698	*RGS12*	1310.16	0.6	0.17	3.34	1.72 × 10^− 2^	57.32 ± 15.26	83.91 ± 16.79
ENSSSCG00000006698	*BCL9*	1518.39	0.6	0.15	3.93	6.26 × 10^− 3^	65.85 ± 11.31	97.82 ± 14.12
ENSSSCG00000032740	*TMEM106B*	1087.56	0.6	0.19	3.03	3.16 × 10^− 2^	47.22 ± 11.83	70.01 ± 17.00
ENSSSCG00000001762	*CIB2*	1555.84	0.6	0.14	3.94	6.12 × 10^− 3^	68.28 ± 20.74	99.43 ± 23.37
ENSSSCG00000039881	*GLI4*	4372.87	0.6	0.11	4.91	4.17 × 10^− 4^	191.55 ± 31.84	279.82 ± 30.26
ENSSSCG00000035875	*OS9*	1461.11	0.5	0.14	4.00	5.97 × 10^− 3^	64.27 ± 18.89	93.23 ± 27.36
ENSSSCG00000035677	*EPOP*	4434.57	0.5	0.16	3.24	2.02 × 10^− 2^	192.35 ± 64.95	285.68 ± 107.63
ENSSSCG00000012952	*TMEM151A*	1087.56	0.5	0.16	3.25	2.01 × 10^− 2^	95.38 ± 28.05	134.85 ± 25.36
ENSSSCG00000015891	*TBR1* ^1^	5918.55	0.5	0.14	3.63	1.07 × 10^− 2^	262.00 ± 79.12	375.99 ± 109.51
ENSSSCG00000000746	*WASHC1* ^2^	1780.84	0.5	0.16	3.27	2.01 × 10^− 2^	80.14 ± 24.17	111.82 ± 18.48
ENSSSCG00000016217	*DNAJB2* ^3^	2408.58	0.5	0.14	3.80	7.93 × 10^− 3^	104.39 ± 26.10	155.25 ± 64.31
ENSSSCG00000001619	*FOXP4* ^4^	1453.92	0.5	0.16	3.12	2.56 × 10^− 2^	64.62 ± 13.22	92.11 ± 19.81
ENSSSCG00000012850	*DEAF1* ^5^	16686.87	0.5	0.12	4.13	4.24 × 10^− 3^	748 ± 181.38	1050.30 ± 159.35

^1^T-box brain transcription factor 1

^2^WASH complex subunit 1

^3^DnaJ heat shock protein family (Hsp40) member B2

^4^Forkhead box P4

^5^DEAF1 transcription factor

**Table 4:** Results from DESeq2 differential expression analysis for the top 15 downregulated genes in sperm samples from six boars at 10-months, compared to 7-months of age (*n* = 12 samples total). Genes are ranked by magnitude of log_2_ fold change (log_2_FC ≤ −0.5). All genes are protein-coding except ENSSSCG00000018063 (Mt_rRNA). The table includes ensembl gene ID, external gene name, base mean normalized values (baseMean), log_2_ fold change (log_2_FC), standard error of the fold change (lfcSE), Wald test statistics (stat) and adjusted *P*-value (adj *P*-value). CPM values were calculated as counts per million mapped fragments from DESeq2 size-factor normalized counts and mean ± SD were calculated within each age group

Ensgene ID	External Gene Name	baseMean	log_2_FC	lfcSE	stat	adj *P*	CPM 7 mo (mean ± SD)	CPM 10 mo (mean ± SD)
ENSSSCG00000018063		3780.19	−0.9	0.16	−5.48	9.67 × 10^− 5^	265.52 ± 163.25	141.97 ± 61.23
ENSSSCG00000014217	*FEM1C*	1686.26	−0.7	0.14	−4.86	4.43 × 10^− 4^	114.45 ± 60.94	67.32 ± 22.77
ENSSSCG00000002535	*HSP90AA1*	1644.41	−0.6	0.19	−3.47	1.44 × 10^− 2^	110.65 ± 65.56	66.61 ± 19.90
ENSSSCG00000008372	*EHBP1*	4150.54	−0.6	0.13	−4.80	5.12 × 10^− 4^	279.77 ± 183.83	167.64 ± 68.84
ENSSSCG00000029249	*NAV3*	5125.87	−0.6	0.12	−5.19	2.44 × 10^− 4^	334.71 ± 71.50	217.84 ± 53.28
ENSSSCG00000006901	*EVI5*	5227.88	−0.6	0.14	−4.29	2.79 × 10^− 3^	338.38 ± 89.50	225.16 ± 69.19
ENSSSCG00000060494	*FSIP2*	2378.55	−0.6	0.13	−4.37	2.03 × 10^− 3^	156.94 ± 77.57	99.46 ± 26.17
ENSSSCG00000007026	*VDAC3*	1578.56	−0.6	0.17	−3.38	1.58 × 10^− 2^	100.25 ± 15.25	69.91 ± 21.40
ENSSSCG00000037977	*ALMS1*	10215.12	−0.5	0.11	−4.91	4.17 × 10^− 4^	657.00 ± 158.63	444.15 ± 87.06
ENSSSCG00000011915	*GRAMD1C*	1048.11	−0.5	0.15	−3.74	9.02 × 10^− 3^	66.70 ± 9.88	46.28 ± 10.76
ENSSSCG00000011117	*UPF2*	1407.68	−0.5	0.15	−3.66	1.01 × 10^− 2^	89.57 ± 19.90	62.17 ± 15.27
ENSSSCG00000008382	*USP34* ^1^	4334.07	−0.5	0.14	−3.60	1.13 × 10^− 2^	280.31 ± 114.94	186.89 ± 37.57
ENSSSCG00000022830	*KANSL1L* ^2^	7569.74	−0.5	0.13	−3.90	6.69 × 10^− 3^	483.97 ± 164.21	332.01 ± 86.56
ENSSSCG00000035379	*JCHAIN* ^3^	1025.87	−0.5	0.17	−2.95	3.73 × 10^− 2^	66.94 ± 39.67	43.64 ± 12.99
ENSSSCG00000035136	*SDCCAG8* ^4^	1286.10	−0.5	0.15	−3.35	1.71 × 10^− 2^	81.70 ± 20.03	56.93 ± 8.03

^1^Ubiquitin specific peptidase 34

^2^KAT8 regulatory NSL complex subunit 1 like

^3^Joining chain of multimeric IgA and IgM

^4^SHH signaling and ciliogenesis regulator SDCCAG8

### Functional enrichment analysis

**Fig. 3:**
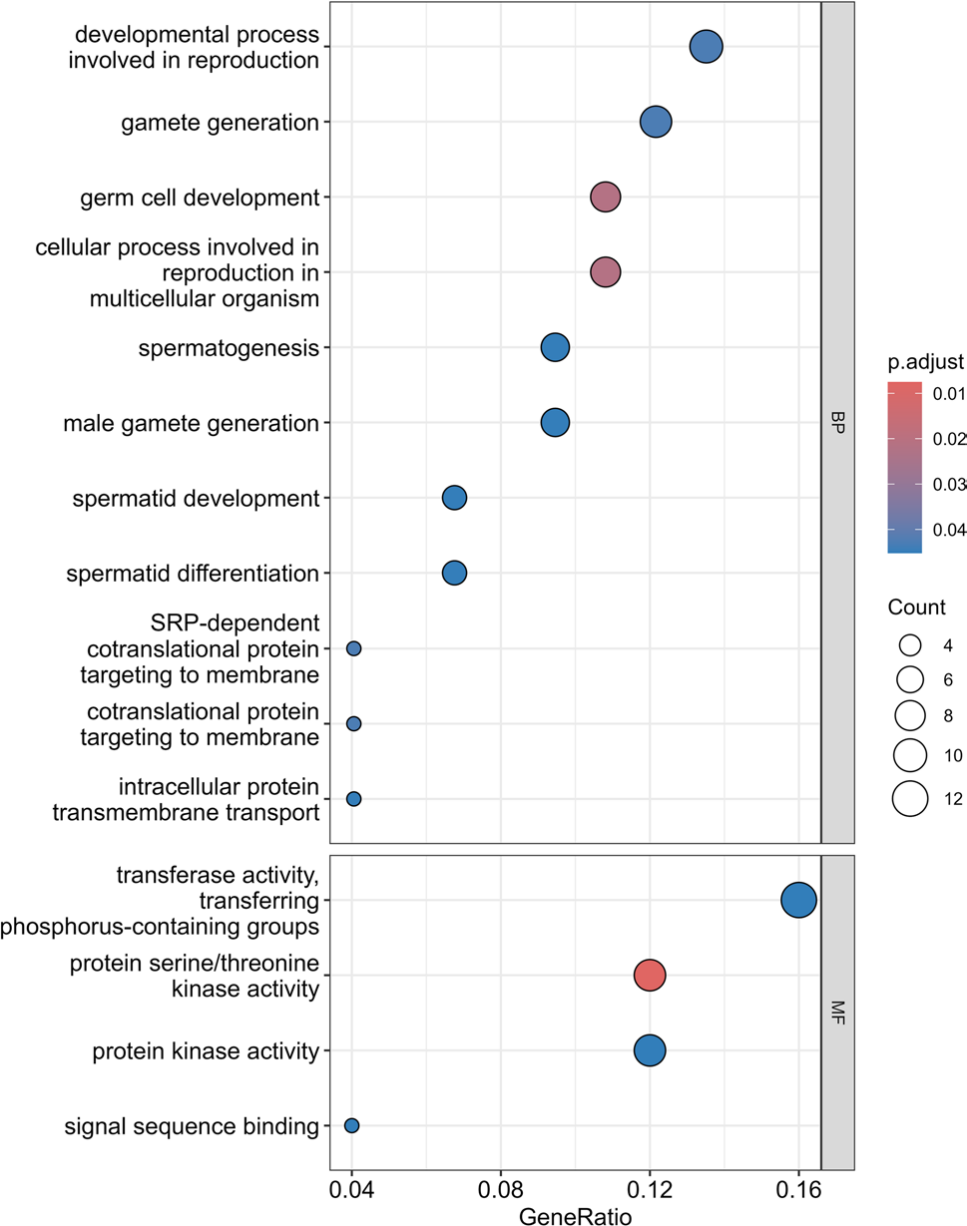
Enriched gene ontology terms among differentially expressed genes in sperm collected from 10-month versus 7-month boars (analysis based on *n* = 6 boars; 12 samples total). The dot plot displays significantly enriched top biological process (BP) and molecular function (MF) terms (adj *P* < 0.05) with gene ratio (proportion of differentially expressed genes belonging to each term) on the x-axis. Dot size represents the number of genes per term, while color represents statistical significance (adj *P*), with red representing more significant values (lower adj *P*-values) and blue representing less significant values (higher adj *P*-values)

To gain insights into the biological significance of the observed transcriptional changes, we performed functional enrichment analyses using GO on DEGs. Our GO analysis revealed significant enrichment of 11 biological processes (adj *P* < 0.05), directly related to reproductive development and four molecular functions being transferase activity, transferring phosphorus-containing groups, protein serine/threonine kinase activity, protein kinase activity and signal sequence binding (Fig. 3, Additional file: Table S5).

Within the biological processes (BP) category, we identified eight significant enrichments of terms directly related to male reproduction (adj *P* < 0.05). The most significantly enriched terms included germ cell development and cellular processes involved in reproduction in multicellular organisms, followed by gamete generation, developmental processes involved in reproduction, spermatogenesis, spermatid development, male gamete generation and spermatid differentiation. We also identified significant enrichment of protein targeting processes, including SRP-dependent cotranslational protein targeting to membrane, cotranslational protein targeting to membrane, and intracellular protein transmembrane transport (adj *P* < 0.05).

Molecular function (MF) analysis revealed significant enrichment of kinase-related activities (adj *P* < 0.05), with protein serine/threonine kinase activity showing the highest significance. Other significantly enriched terms (adj *P* < 0.05) included protein kinase activity, signal sequence binding and transferase activity transferring phosphorus-containing groups.

**Fig. 4:**
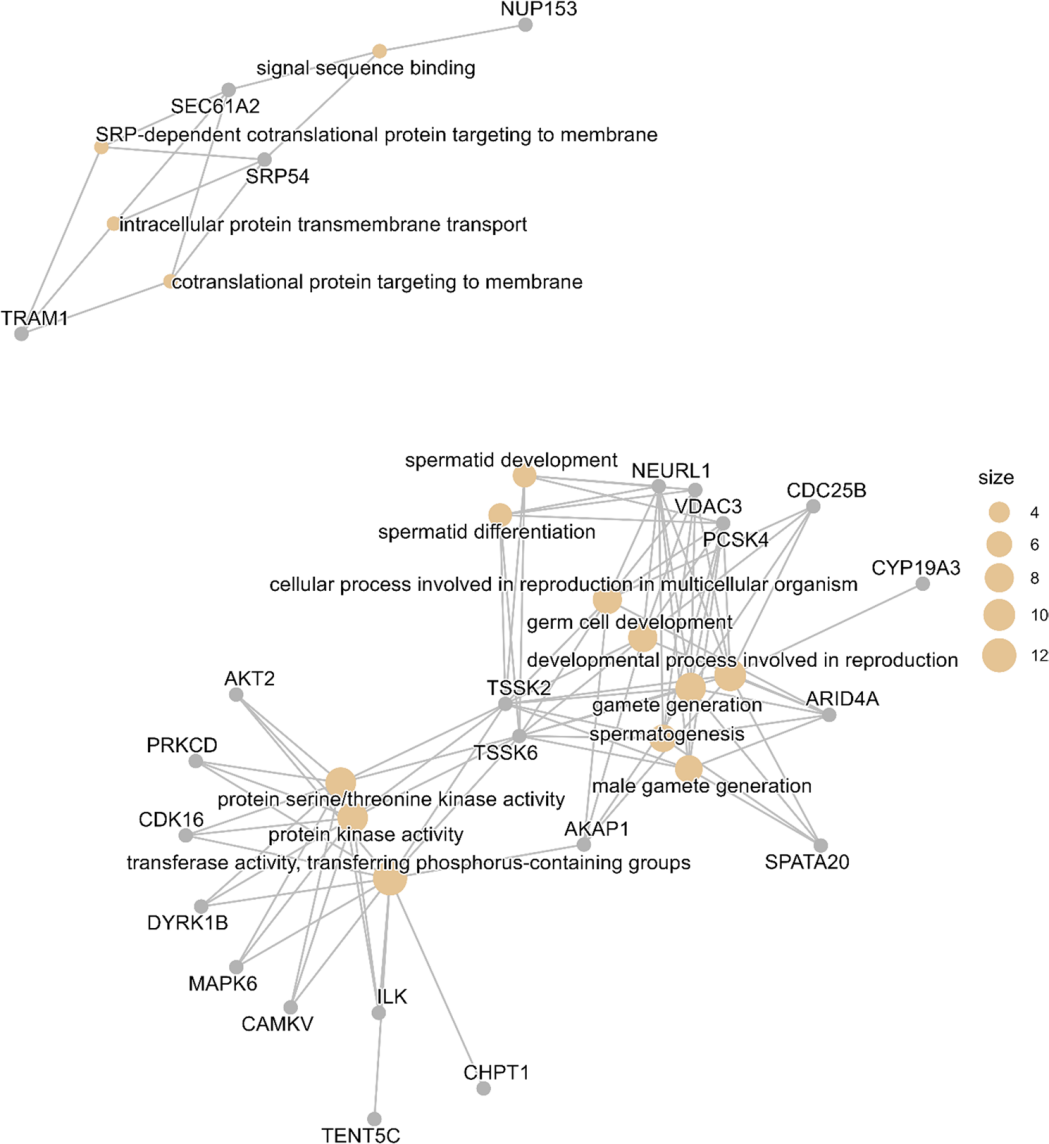
Gene-concept network analysis of differentially expressed genes in sperm of 10-month old versus 7-month old boars (analysis based on *n* = 6 boars; 12 samples total). This gene-concept network plot (cnetplot) depicts the relationships between differentially expressed genes (grey nodes) and enriched Gene Ontology (GO) terms (colored nodes). The size of GO term nodes indicates the number of associated genes. There are three distinct functional clusters: the upper cluster represents membrane protein transport pathways, the central cluster represents reproductive development processes, and the lower cluster represents kinase signaling networks. Several genes like *TSSK2*, *TSSK6*, and *AKAP1* connect the two clusters. Line connections represent associations between GO terms and genes based on GO annotations

Our network analysis also indicates distinct yet interconnected functional clusters (Fig. 4). Notably, the connection between kinase activity and structural processes, specifically spermatid development, is mediated by *TSSK2*, *TSSK6*, and *AKAP1*. This interconnection demonstrates how regulatory networks orchestrate the functional optimization of sperm as boars transition from puberty to sexual maturity.

### Differential miRNA expression during sexual maturation

Following total RNA profiling, we examined the miRNA landscape in boar sperm samples to understand the small RNA regulatory network during sexual maturation. Our miRNA-Seq analysis of the 12 sperm libraries yielded an average of 28.93 ± 4.04 million reads per sample with an average length of 83 bp. Quality filtering retained 54.62% ± 3.03% of reads, with 63.13% ± 3.17% of retained reads were successfully assigned to known small RNA categories, with 19.97% ± 4.04% mapping specifically to mature miRNA (Additional File: Table S6). From this dataset, we detected on average 377 ± 10 mature miRNAs per library. After applying our standard expression filter (at least 10 read counts in a minimum of 6 samples), 240 miRNAs were retained for downstream differential expression analysis. The five most abundant miRNAs identified across all samples were ssc-miR-16, ssc-miR-191, ssc-let-7a, ssc-let-7i-5p, and ssc-miR-30d.

**Fig. 5:**
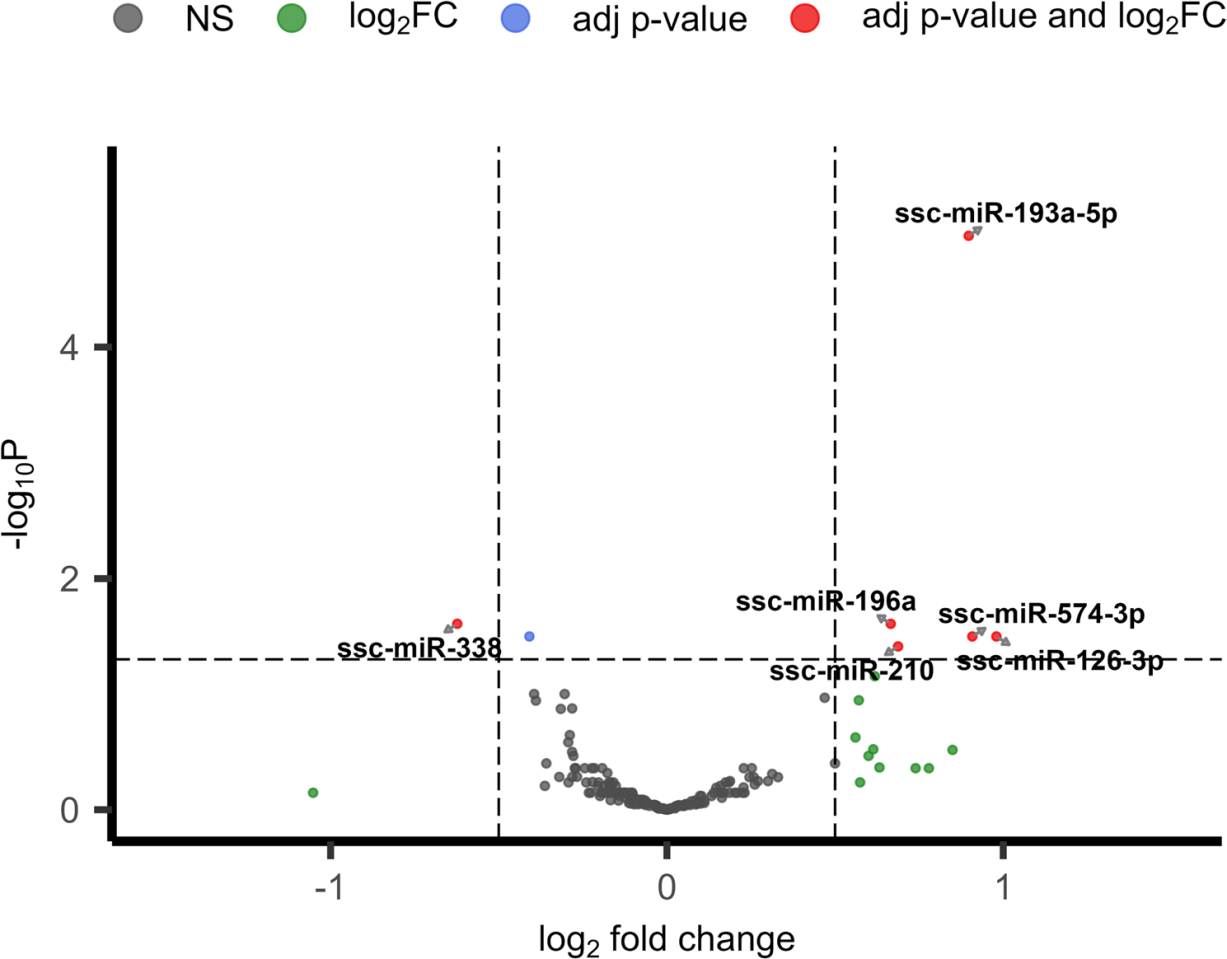
Differential expression of miRNAs in sperm collected from six Duroc boars (*n* = 12 samples total) at 7-months and 10-months of age. Volcano plot displaying log_2_ fold change (x-axis) versus statistical significance (-log_10_ adj *P*-value, y-axis) for 179 miRNAs. Red dots represent miRNAs meeting both significance (adj *P* < 0.05) and fold change (|log_2_FC| ≥ 0.5) thresholds and are labelled. Blue dots indicate miRNAs with significant *P*-values (adj *P* < 0.05) but insufficient fold change (|log_2_FC| < 0.5, and green dots show miRNAs with substantial fold change (|log_2_FC| ≥ 0.5) but lacking statistical significance (adj *P* > 0.05). Grey dots show miRNAs which are non-significant (NS)

Differential expression analysis identified six significantly altered miRNAs between pubertal and mature boars (adj *P* < 0.05, |log_2_FC| ≥ 0.5), with five upregulated and one downregulated miRNA in mature boar samples, compared to pubertal boar samples (Fig. 5; Table 5). ssc-miR-193a-5p showed the highest statistical significance among upregulated miRNAs (log_2_FC = 0.9, adj *P* = 1.09 × 10^− 5^), while ssc-miR-126-3p displayed the greatest fold change (log_2_FC = 1.0). The remaining upregulated miRNAs, ssc-miR-196a, ssc-miR-574-3p, and ssc-miR-210 showed moderate but significant increases, while ssc-miR-338 was downregulated in mature boars.

**Table 5:** Differentially expressed miRNAs in sperm collected from six boars at 7-months and 10-months of age (*n* = 12 samples total). Positive log_2_FC values denote higher expression in sperm in mature boars (upregulated), and negative values denote lower expression in sperm in mature boars (downregulated). The table includes miRNA ID, base mean normalized values (baseMean), log_2_ fold change (log_2_FC), standard error of the fold change (lfcSE), Wald test statistics (stat), and adjusted *P*-value (adj *P*-value). CPM values were calculated as counts per million mapped fragments from DESeq2 size-factor normalized counts and mean ± SD were calculated within each age group

	miRNA	baseMean	log_2_FC	lfcSE	stat	adj *P*	CPM 7 mo (Mean ± SD)	CPM 10 mo (Mean ± SD)
Upregulated	ssc-miR-193a-5p	230.21	0.9	0.16	5.39	1.09 × 10^− 5^	44.53 ± 26.6	80.69 ± 37.57
	ssc-miR-196a	846.34	0.7	0.19	3.49	2.46 × 10^− 2^	187.32 ± 155.9	273.03 ± 186.27
	ssc-miR-126-3p	332.48	1	0.29	3.34	3.17 × 10^− 2^	78.91 ± 92.39	101.94 ± 105.95
	ssc-miR-574-3p	187.21	0.9	0.27	3.31	3.17 × 10^− 2^	43.27 ± 50.13	58.56 ± 51.6
	ssc-miR-210	674.89	0.7	0.21	3.16	3.87 × 10^− 2^	136.92 ± 90.81	230.17 ± 179.71
Downregulated	ssc-miR-338	177.17	−0.6	0.18	−3.62	2.46 × 10^− 2^	59.22 ± 25.08	37.06 ± 11.23

### miRNA target prediction and functional enrichment analysis

Our target prediction analysis revealed extensive regulatory potential for the differentially expressed miRNAs in mature boar sperm. Among the upregulated miRNAs, ssc-miR-126-3p targeted 1187 genes, followed by ssc-miR-193a-5p (821 targets) and ssc-miR-574-3p (752 targets) (Additional file: Table S7). Collectively, these three miRNAs were predicted to regulate a network of 2459 unique genes, suggesting widespread post-transcriptional regulation during sexual maturation. We observed overlap between the predicted target genes of these three miRNAs. Specifically, ssc-miR-126-3p and ssc-miR-193a-5p shared 109 common predicted targets, while ssc-miR-126-3p and ssc-miR-574-3p shared 117 predicted targets. Additionally, ssc-miR-193a-5p and ssc-miR-574-3p converged on 100 common predicted genes. Most notably, 25 genes were predicted to be targeted by all three upregulated miRNAs. This pattern of overlapping regulation suggests potential coordinated control of key developmental pathways during sperm maturation.

**Fig. 6:**
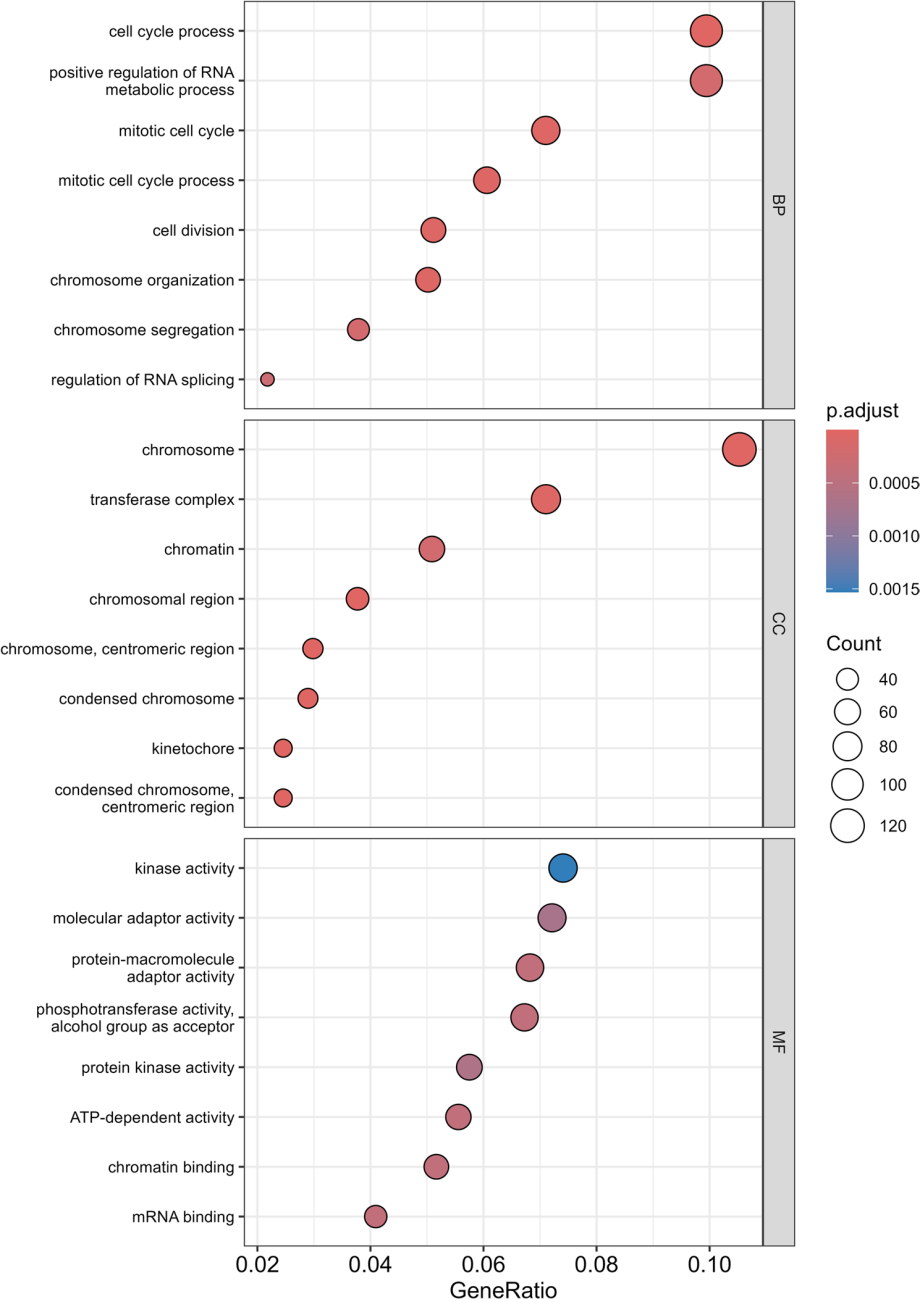
Gene ontology enrichment analysis of predicted target genes for differentially expressed miRNAs in sperm collected from boars at 7-months and 10-months of age (analysis based on *n* = 6 boars; 12 samples total). Top enriched biological process (BP), molecular function (MF), and cellular component terms (adj *P* < 0.05) are shown with gene ratio (proportion of target genes in each term) on the x-axis. Dot size represents the number of genes per term, while color represents statistical significance (adj *P*), with red representing more significant values (lower adj *P*-values) and blue representing less significant values (higher adj *P*-values)

GO enrichment analysis of the miRNA targets identified 232 significantly enriched terms (Biological Processes = 167, Molecular Function = 36, Cellular Component = 29) (adj *P* < 0.05) (Fig. 6, Additional file: Table S8). Among biological processes, cell cycle processes (adj *P* = 3.69 × 10⁻^7^), mitotic cell cycle processes (adj *P* = 2.62 × 10⁻^5^), and the mitotic cell cycle (adj *P* = 2.62 × 10⁻^5^) were significant. For molecular functions, mRNA binding (adj *P* = 3.03 × 10^− 6^), chromatin binding (adj *P* = 4.15 × 10⁻^4^), and protein-macromolecule adaptor activity (adj *P* = 4.15 × 10⁻^4^) were significant, suggesting active roles of these miRNAs in mRNA and chromatin organization during maturation. Cellular components related to chromosome structure and function were also significant, including chromosome (adj *P* = 1.14 × 10⁻^13^), chromosomal region (adj *P* = 3.77 × 10⁻^9^), and chromosome, centromeric region (adj *P* = 3.77 × 10⁻^9^).

### Integrated miRNA-mRNA analysis

**Fig. 7:**
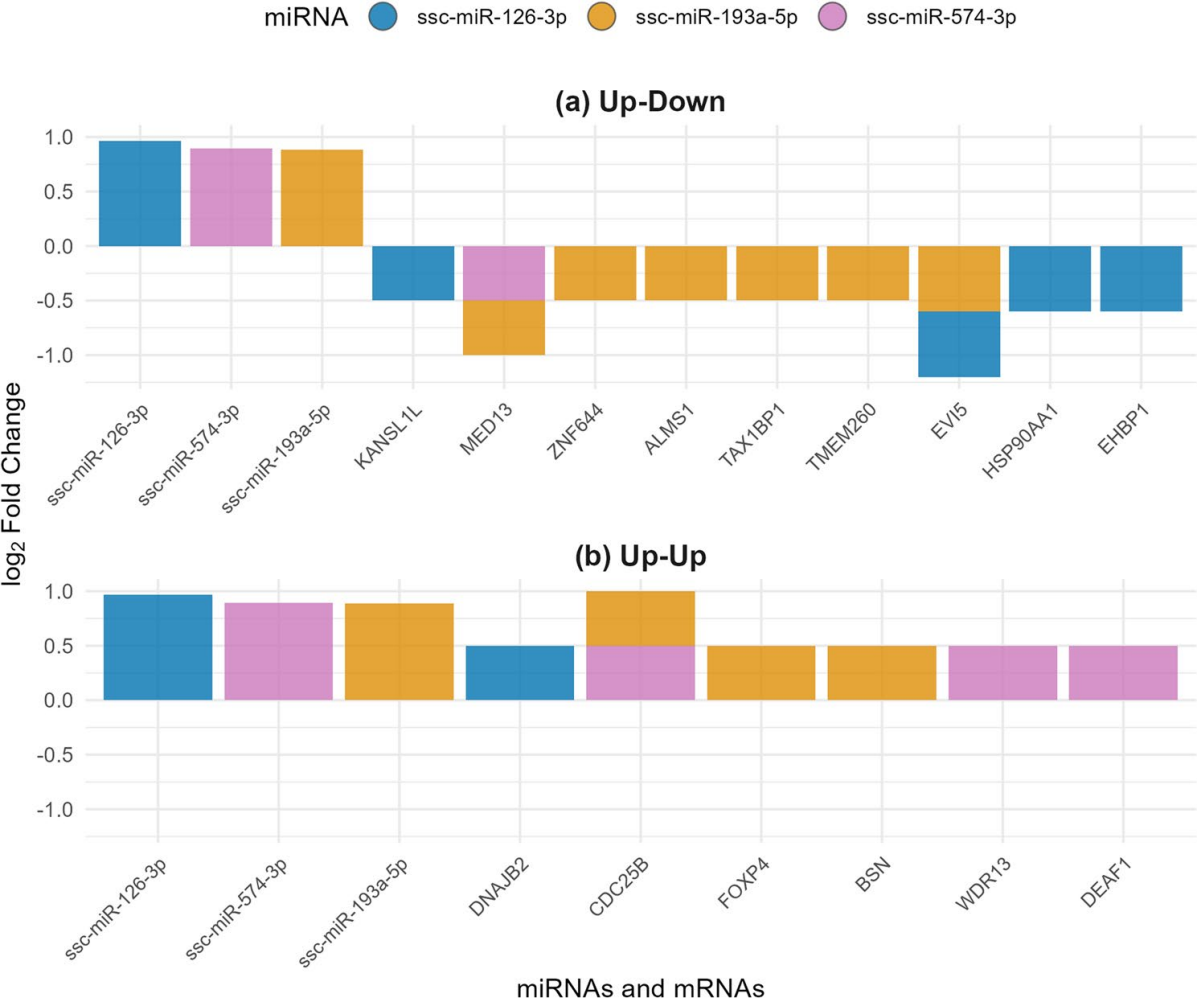
miRNA-mRNA regulatory relationships in sperm collected from six Duroc boars (*n* = 12 samples total) at 7-months and 10-months of age. Bar plots displaying log_2_ fold change expression patterns for miRNAs and their predicted target mRNAs. Panel (a) shows inverse pairs, upregulated miRNAs and their downregulated target mRNAs. Panel (b) displays concordant pairs, upregulated miRNAs with their upregulated target mRNAs. Colors indicate different miRNA regulatory networks: blue bars represent ssc-miR-126-3p and its targets, orange bars represent ssc-miR-193a-5p and its targets, and purple bars represent ssc-miR-574-3p and its targets. miRNAs are positioned first (left side of each panel), followed by their target mRNAs. Only differentially expressed genes meeting both significance (adj *P* < 0.05) and fold change (|log_2_FC| ≥ 0.5) thresholds are shown

To identify functionally relevant miRNA-mRNA interactions during sexual maturation, we performed an integrated analysis of our miRNA and mRNA datasets by intersecting predicted miRNA targets with our differentially expressed mRNAs (adj *P* < 0.05 & |log_2_FC| ≥ 0.5). This analysis identified 11 and seven miRNA-mRNA pairs with negatively (upregulated miRNA with downregulated mRNA) and positively (upregulated miRNA with upregulated mRNA) correlated expression, respectively. These miRNAs and mRNAs were differentially expressed between pubertal and mature boar samples. (Fig. 7).

Three miRNAs dominated these regulatory interactions: ssc-miR-193a-5p, ssc-miR-126-3p, and ssc-miR-574-3p. The most significantly upregulated miRNA, ssc-miR-193a-5p (Table 5), showed negative expression relationships with six downregulated mRNAs in our dataset among its predicted targets, including mediator complex subunit 13 (*MED13*), zinc finger protein 644 (*ZNF644*), *ALMS1*, tax1 binding protein 1 (*TAX1BP1*), and transmembrane protein 260 (*TMEM260*). Additionally, ssc-miR-193a-5p displayed positive expression with three upregulated predicted targets: *CDC25B*, *FOXP4*, and bassoon presynaptic cytomatrix protein (*BSN*). Similarly, ssc-miR-126-3p showed negative expression relationships with four downregulated mRNAs, including *EVI5*, *HSP90AA1*, *EHBP1*, and KAT8 regulatory NSL complex subunit 1 like (*KANSL1L*) while exhibiting a positive relationship with only *DNAJB2*. The third upregulated miRNA, ssc-miR-574-3p, displayed a negative relationship with one downregulated mRNA (*MED13*) and positive relations with three upregulated predicted targets (*CDC25B*, WD repeat domain 13 (*WDR13*), and *DEAF1*).

Notably, several genes were targeted by multiple upregulated miRNAs, suggesting coordinated regulation. For instance, *EVI5* was predicted as a target for both ssc-miR-126-3p and ssc-miR-193a-5p, while *MED13* was predicted to be targeted by both ssc-miR-193a-5p and ssc-miR-574-3p. This convergence of multiple miRNAs on common targets highlights potential synergistic regulation of key genes during sexual maturation.

## Discussion

Our study presents the first comprehensive characterization of transcriptomic changes in boar sperm during the transition from puberty to sexual maturity. Using paired sperm samples collected from the same Duroc boars at approximately 7 and 10 months of age with combined total RNA-Seq and miRNA-Seq analysis, we identified distinctive transcriptional signatures that characterize this important reproductive transition. Our findings reveal a predominant pattern of upregulation in both protein-coding genes and miRNAs, along with coordinated mRNA-miRNA pairs that collectively regulate sperm maturation in Duroc boars.

Our total RNA-Seq sequencing depth (30.41 ± 4.83 million reads per end) and post-trimming clean read counts (29.01 ± 4.87 million) are robust and compare favorably with the existing boar sperm literature [16, 19, 20, 47]. Furthermore, our overall mapping rate of 78.08% ± 3.06% to the *Sus scrofa* genome is consistent with previous reports [16, 20]. Thus, our sequencing metrics were of high quality for downstream analysis. While we observed a high Picard duplication rate, this is expected in preparing RNA-Seq libraries from low-input, highly fragmented RNA, which is characteristic of spermatozoa and is comparable to the study by Gòdia et al. [16]. The most abundant coding genes we identified in our dataset across all samples, including *PRM1*, *OAZ3* and *HSPB9* were also among the most abundant protein-coding genes in boar sperm in other studies [11, 15, 16]. This is biologically significant as these genes are reported to be mainly associated with sperm functionality and the process of embryonic development [14].

Similarly, for our small RNA-Seq analysis, we obtained greater sequencing depth (28.93 ± 4.04 million raw reads) in fresh boar sperm as compared to the previous study (18.96 million raw reads) by Dai et al. [19], confirming sufficient depth for miRNA profiling. The average of 377 miRNAs we retained for analysis is considerably higher than the 259 known miRNAs reported previously by the same study [19]. This enhanced detection likely reflects our greater sequencing depth and updated miRNA annotations. Likewise, the most abundant miRNAs we identified in our dataset, miR-16, miR-191, miR-30d, miR-191, let-7a are consistently cited as the most dominant miRNAs in boar sperm [11, 16, 18]. The high prevalence of these miRNAs is functionally relevant. let-7 family (let-7a, let-7i) members are associated with spermatogenesis, motility, and sperm morphology [21]. miR-191 has been associated with improved development of embryos in humans [48]. miR-30d has been identified to be differentially expressed in human populations with poor sperm parameters, including asthenozoospermia, teratozoospermia and oligoazoospermia [49].

### Transcriptomic landscape transformation

The most striking feature of the transcriptomic landscape during sexual maturation is the predominant increase in RNA abundance, with 65% of DEGs showing higher levels in mature compared to pubertal boars. This change towards increased RNA levels suggests that sexual maturation is characterized by larger gene activity during spermatogenesis. Hierarchical clustering analysis showed a clear separation of samples by age, establishing a reproducible molecular signature of maturity that transcends individual variation. Notably, two boars appeared as outliers in the clustering analysis; this observation may reflect individual biological variation in achieving sexual maturity. It is biologically plausible that one boar matured slightly faster (its 7 month sample resembled a mature state) and one matured slightly slower (its 10-month sample still resembled a pubertal state). While previous studies have examined transcriptomic profiles of sperm of sexually mature animals across different species, such as boars [13], bulls [50], rams [51], and stallions [52], our work uniquely captures the dynamic changes occurring during the developmental window from puberty to sexual maturity in Duroc boars.

### Enhanced sperm function through protein-coding gene modulation

Our analysis identified *NCLN* as the top differentially expressed upregulated gene in mature boars. *NCLN* encodes nicalin, a protein that functions as a component of the multi-pass translocon complex that mediates insertion of membrane proteins into lipid bilayers [53]. This significant upregulation suggests fundamental changes in membrane protein organization during sperm maturation. Several other prominently upregulated genes appear to support multiple aspects of sperm functionality. For instance, *RGS12*, coding for a regulator of G protein signaling, is involved in Ca^2+^ oscillation required for oocyte activation after fertilization in humans [54]. Similarly, *CIB2* produces a calcium and integrin binding family member that regulates intracellular calcium homeostasis [55] and has been implicated in mouse spermatogenesis [56]. In yak, *CIB2* transcripts were hypermethylated at m^6^A sites in the adult testis compared to the juvenile testis, contributing to spermatogenesis [57], supporting our findings for sexual maturity.

Additionally, we observed the significant upregulation of genes related to chromatin remodeling and gametogenesis, including *FOXP4* and *PHC1* [58, 59]. Transcript levels of *FOXP4* are reported to increase during the development of spermatogonial stem cells, promoting their proliferation and supporting normal sperm production [58]. A recent study by Pértille et al. [60] identified another FOX member, forkhead box I1 (*FOXI1*), as one of the key genes associated with differentially methylated regions that distinguish high- and low-fertility boars. Taken together, these findings suggest that the FOX gene family may be an important regulator of male fertility in pigs. *PHC1* produces a protein that acts as a central regulator of differentially expressed genes in mouse spermatogonial stem cells, maintaining balance between self-renewal and differentiation during spermatogenesis [59]. PHC1, which has been reported to be expressed in spermatocytes [61], is a component of Polycomb repressive complex (PRC1), which mediates histone H2A monoubiquitination and chromatin remodelling [62]. In mouse testes, PRC1 is reported to be linked to the maintenance of undifferentiated spermatogonia, progression of spermatocytes through meiotic prophase, and spermatogenic differentiation [63]. Additionally, *PRC1* is identified as an upregulated gene in aged human oocytes [64]. This suggests its role as a regulator of gamete viability. Our analysis also revealed significant upregulation of other genes critical for sperm functionality during sexual maturation. *CDC25B* encodes the CDC25B protein, which is found to localize to the midpiece, principal piece, and head cytosol of human spermatozoa, suggesting a role in non-cycle functions in terminally differentiated germ cells [65]. AKAP1 anchors protein kinase A to the outer mitochondrial membrane, facilitating localized phosphorylation events that support mitochondrial energy production and sperm motility in human spermatozoa [66]. Levels of *AKAP1* transcripts were significantly higher in normozoospermic men than in those with reproductive disorders [67] and moreover, *Akap1* has been linked to spermatogenesis in mice [68]. The coordinated upregulation of these genes during sexual maturation suggests enhanced regulation of energy metabolism, phosphorylation cascades, and chromatin organization as boars transition from puberty to sexual maturity.

### Downregulation of developmental and metabolic genes during sexual maturation

We observed significant downregulation of *HSP90AA1* in our study, a gene that encodes a molecular chaperone involved in protein maturation and regulation [69]. Interestingly, previous studies have found HSP90AA1 protein to be overrepresented in low-fertility bull sperm, suggesting that its downregulation in mature boar sperm may be beneficial for optimal functionality. HSP90AA1 has also been implicated in regulating the acrosome reaction in yaks [70]. Therefore, we speculate that the downregulation of *HSP90AA1* in our study could represent a finely tuned optimization of protein folding and stability required for mature boar sperm function.

Our analysis also revealed that one mitochondrial rRNA was significantly downregulated in mature boars. It may indicate the remodeling of energy production pathways during maturation [71], however our results do not reflect a global downregulation of mitochondrial gene products. FSIP2, a protein associated with the sperm fibrous sheath and specific to spermatogenic cells [72], showed decreased expression in sperm from mature boars in our study. FSIP2 has been identified in the phosphoproteomics studies in yak as part of a protein network with AKAP3, AKAP4, and CABYR, suggesting roles in protein kinase A binding, cytoskeletal organization, and sperm motility [73]. Mouse models with *fsip2* mutations demonstrate multiple morphological abnormalities of sperm flagella, while its overexpression produces elongated tail structures and altered acrosome [72]. This suggests that precise regulation of FSIP2 levels is critical for proper sperm development. The downregulation observed herein for mature boars could reflect a developmental fine-tuning, where lower levels are optimal for the maintenance of properly formed flagellar and acrosomal structures in fully matured sperm. 

*VDAC3* encodes the voltage-dependent anion channel 3 protein, which localizes to the acrosomal region and midpiece of spermatozoa. In mouse models, VDAC3 protein deficiency caused significant decrease in sperm motility, viability, acrosome reaction, capacitation, fertilization, and embryo development. Proper regulation of VDAC3 is essential for proper mitochondrial sheath formation during spermatogenesis, sperm function and male fertility [74, 75]. Similarly, upregulation of gene family member *VDAC1* was reported in Duroc boars with high DNA fragmentation index [76]. The downregulation of *VDAC3* in the sperm of 10-months old in contrast to the 7-month-old samples in our study may indicate fine-tuning of mitochondrial dynamics as sperm reach functional maturity. *ALMS1* was also significantly downregulated in the sperm of 10-months old individuals. Loss of ALMS1 protein function in mouse resulted in primary spermatogenic defects characterized by disrupted progression from round to elongating spermatids, abnormal sperm tail and mitochondrial structure, and testicular atrophy leading to male infertility [77]. Likewise, conditional knockout of *Upf2* in mouse germ cells causes severe spermatogenic defects and male infertility, highlighting *UPF2*’s important role in fertility [78].

### Functional integration through biological pathways

The GO enrichment analysis suggests possible biological functions or pathways associated with the observed transcriptomic changes occurring during boar maturation. As ejaculated sperm already carries a highly specialized transcriptome enriched for sperm and reproduction related function, many genes that are not differentially expressed are also likely annotated to reproductive GO terms. We therefore interpret the GO results by focusing on the specific DEGs that drive the enriched terms.

The significant enrichment of the spermatid development and differentiation terms suggests that even in ejaculated sperm, the transcriptomic signature of developmental processes remains detectable and undergoes dynamic regulation from puberty to sexual maturity. Several genes driving this enrichment were common across multiple reproduction-related GO terms, including testis specific serine kinase 2 (*TSSK2)*, testis specific serine kinase 6 (*TSSK6)*, *AKAP1*, spermatogenesis associated 20 (*SPATA20*), AT-rich interaction Domain 4 A (*ARID4A*), cytochrome P450 family 19 subfamily A member 3 (*CYP19A3*), *CDC25B*, proprotein convertase subtilisin/kexin type 4 (*PCSK4*), *VDAC3*, and neuralized E3 ubiquitin protein ligase 1 (*NEURL1*) (Fig. 4). Notably, many of these genes have established roles in sperm function that we have previously discussed in this paper. For instance, the upregulation of *AKAP1* and *CDC25B* in sperm of 10-months old boars likely supports mitochondrial function and non-cell cycle functions, respectively [65, 66] while the downregulation of *VDAC3* might represent a refinement in mitochondrial dynamics during spermatogenesis [75].

The identification of protein targeting to the membrane as an enriched process, represented by signal recognition particle 54 (*SRP54*), Sect. 61 translocon subunit alpha 2 (*SEC61A2*), and translocon associated membrane protein 1 (*TRAM1*) highlights the importance of proper protein localization in mature sperm. Membrane protein organization is crucial for acrosome reaction, and sperm-egg fusion, and our findings suggest that these processes undergo refinement during sexual maturation. Our analysis also revealed significant enrichment of protein kinase activity terms, driven by a diverse set of genes including AKT serine/threonine kinase 2 (*AKT2*), protein kinase C delta (*PRKCD*), cyclin dependent kinase 16 (*CDK16*), dual specificity tyrosine phosphorylation regulated kinase 1B (*DYRK1B*), mitogen-activated protein kinase 6 (*MAPK6*), CaM kinase like vesicle associated (*CAMKV*), integrin linked kinase (*ILK*), choline phosphotransferase 1 (*CHPT1*), terminal nucleotidyltransferase 5 C (*TENT5C*), *TSSK2*, *TSSK6*, and *AKAP1*. The enrichment of this functional category underscores the importance of phosphorylation-dependent signaling in regulating sperm maturation and function.

Collectively, these findings provide a systems-level perspective on boar sperm maturation. These GO based observations highlight specific developmental, membrane associated and signaling modules, and the underlying DEGs, that appear to be adjusted as boars progress from puberty to sexual maturity.

### miRNA regulation during sexual maturation

Beyond protein-coding genes, our study revealed significant changes in miRNA expression during sexual maturation. Five out of six differentially expressed miRNAs were upregulated in mature boars, mirroring the trend observed in protein-coding genes. Among the upregulated miRNAs, we found ssc-miR-193a-5p, which has been previously implicated in sperm function across multiple species. In bovine studies, miR-193a-3p was overexpressed in both sperm and seminal plasma of high-fertility bulls, directly connecting this miRNA to reproductive success [79]. Human literature further supports this connection, identifying miR-193a as a key regulator in asthenozoospermia influencing sperm motility [80].

ssc-miR-196a has been identified as evolutionarily conserved between amphibians and mammals, critical for spermatogenesis across all tetrapods, spanning approximately 350 million years, and misregulation of this miR-196 contributes to male sterility [81]. Intriguingly, Lian et al. [82] found miR-196 expression restricted to immature porcine testes (1 month), contrasting with our finding of increased expression in sperm of mature vs. pubertal boars. This apparent discrepancy may reflect the different regulatory roles of miR-196 during testicular development versus sperm maturation. Zhang et al. [83] demonstrated that miR-196a enhances Sertoli cell proliferation and reduces apoptosis by targeting regulator of chromosome condensation 2 (*RCC2*) and ATP binding cassette subfamily B member 9 (*ABCB9*) genes in pigs, suggesting its importance in establishing optimal cellular environments for sperm production.

The ssc-miR-126-3p has been extensively characterized in reproductive contexts across species. In pigs, miR-126 has been reported to stimulate cell proliferation and suppress apoptosis in immature Sertoli cells targeting *PIK3R2* and activating the PI3k/AKT signaling pathways [84]. In humans, reduced miR-126 expression is documented in patients with a complete absence of sperm due to impaired spermatogenesis [85].

The upregulation of ssc-miR-574-3p connects directly to energy metabolism regulation. Ma et al. [86] demonstrated that miR-574 directly targets mt-ND5, a component of the mitochondrial respiratory chain complex I, thereby regulating mitochondrial function and ATP generation in human sperm. Elevated levels of miR-210 have been observed in testicular tissues with maturation arrest/hypospermatogenesis and in the seminal plasma of varicocele patients [87, 88]. These findings underscore the context-dependent roles of miR-210, with its upregulation in sperm from mature boars potentially reflecting a specific regulatory adaptation during sexual maturity.

The downregulation of ssc-miR-338 represents an equally important aspect of the sexual maturation process. Madison-Villar and Michalak [81] identified miR-338 among their evolutionarily conserved set of miRNAs critical for spermatogenesis across tetrapods, with misexpression correlating with testicular dysfunction in sterile fish hybrids. Our analysis further suggested that three miRNAs: ssc-miR-193a-5p, ssc-miR-126-3p, and ssc-miR-574-3p, emerged as central regulators, collectively targeting 2,459 unique genes.

### Integrative analysis of miRNA-mRNA interactions

Integrated analysis was done by intersecting differentially expressed miRNAs and their predicted mRNA targets that were in our datasets. The convergence of multiple upregulated miRNAs on common targets suggests redundant control mechanisms ensuring proper sexual maturation. It might also indicate a fine-tuning of gene expression or cooperative effects of the miRNAs. For instance, *EVI5* was predicted to be targeted by both ssc-miR-126-3p and ssc-miR-193a-5p *MED13* and CDC25B were predicted to be targeted by both ssc-miR-193a-5p and ssc-miR-574-3p.

Our analysis identified six target genes that were downregulated and three target genes upregulated by ssc-miR-193a-5p, including *EVI5*, *ALMS1*, and *CDC25B.* Proteins encoded by *EVI5* are involved in cell cycle progression [89], ALSM1 protein has a role in spermatogenesis, sperm tail, and mitochondrial structure in mice [77], and CDC25B protein localizes to the human sperm midpiece and head [65]. This suggests that ssc-miR-193a-5p coordinates the fine-tuning of both structural and functional components of sperm.

Similarly, upregulated ssc-miR-126-3p potentially targeted one upregulated gene and four downregulated genes in our study, including *EVI5*, and HSP90AA1. Overexpression of HSP90AA1 protein is linked to poor fertility in bulls [90] and it has also been implicated in regulating the acrosome reaction in yaks [70] and protein folding in human [69]. The downregulation of *HSP90AA1* in our sperm samples from mature boars may therefore represent a finely tuned optimization of protein folding and stability required for mature sperm function. Taken together, our miRNA findings complement and extend the mRNA results. Concurrently, they provide a deeper understanding of the molecular mechanisms driving the transition from puberty to sexual maturity in Duroc boars.

### Limitations and future directions

Our sample size of six boars and focus on a single breed, though adequate for detecting major transcriptomic shifts, may have limited our ability to identify more subtle changes with lower effect sizes and applicability to other breeds. We applied a lower fold-change cutoff (|log_2_FC| ≥ 0.5) than typically used in somatic tissue studies, since mature spermatozoa carry 600 times less RNAs than somatic cell [91] and even modest expression changes can be biologically meaningful during the maturation process.

Future studies can expand our findings in several directions. Comparing RNA levels with quantitative semen traits in a larger sample size could provide further insights into the potential roles of these genes in sexual maturation in sperm. Confirmation of the top 15–20 differentially expressed genes with RT-qPCR on larger cohorts across multiple swine breeds to validate these transcriptomic signatures would enhance their applicability as biomarkers of sexual maturity. Moreover, integrating additional omics data, including metabolomics, proteomics, and epigenetics, would offer a more comprehensive understanding of the regulatory networks governing sexual maturation.

## Conclusion

This study provides the first comprehensive transcriptomic profile of sperm between pubertal (~ 7 months) and mature (~ 10 months) boars. Our paired analysis revealed predominantly upregulated genes during maturation, including *NCLN*, *RGSL2*, *CIB2*, *FOXP4*, *PHC1*, *CDC25B*, and *AKAP1* involved in membrane organization, calcium homeostasis, protein kinase signaling, and reproductive processes. Concurrently, genes associated with sperm development and metabolism, such as *HSP90AA1*,* EVI5*,* FSIP2*,* VDAC3*, and *ALMS1* were downregulated during maturation. We identified six differentially expressed miRNAs and predicted 18 miRNA-mRNA regulatory pairs. Functional enrichment analysis identified biological processes such as reproduction, cell cycle regulation, and chromatin organization, indicating coordinated transcriptional regulation during sexual maturation.

These findings advance our understanding of boar sexual maturation and provide a first step towards potential biomarkers for assessing reproductive development, with practical implications for swine breeding programs where genomic selection drives earlier introduction of young boars.

## Supplementary Information

Supplementary Material 1.

Supplementary Material 2.

Supplementary Material 3.

Supplementary Material 4.

Supplementary Material 5.

Supplementary Material 6.

Supplementary Material 7.

Supplementary Material 8.

Supplementary Material 9.

## Data Availability

The datasets used and/or analyzed during the current study have been deposited in NCBI’s Gene Expression Omnibus and are accessible through GEO Series number GSE305494 (https:/www.ncbi.nlm.nih.gov/geo/query/acc.cgi?&acc=GSE305494).

## Abbreviations

*ABCB9*: ATP binding cassette subfamily B member 9
AI: Artificial insemination
*AKAP1*: A-kinase anchoring protein 1
*AKT2*: AKT serine/threonine kinase 2
*ALMS1*: ALMS1 centrosome and basal body associated protein
*ARID4A*: AT-rich interaction Domain 4 A
BAM: Binary alignment/map
*BCL9*: BCL9 transcription coactivator
BP: Biological processes
*BSN*: Bassoon presynaptic cytomatrix protein
*CAMKV*: CaM kinase like vesicle associated
*CATSPERG*: Cation channel sperm associated auxiliary subunit gamma
*CDC25B*: Cell division cycle 25B
*CDK16*: Cyclin dependent kinase 16
*CHPT1*: Choline phosphotransferase 1
*CIB2*: Calcium and integrin binding family member 2
*CYP19A3*: Cytochrome P450 family 19 subfamily A member 3
*DEAF1*: DEAF1 transcription factor
DEGs: Differentially expressed genes
*DNAJB2*: DnaJ heat shock protein family (Hsp40) member B2
*DNAJB8*: DnaJ heat shock protein family member B8
*DYRK1B*: Dual specificity tyrosine phosphorylation regulated kinase 1B
*EHBP1*: EH domain binding protein 1
*EPOP*: Elongin BC and polycomb repressive complex 2 associated protein
ESTs: Expressed sequence tags
*EVI5*: Ectropic viral integration site 5
*FEM1C*: Fem-1 homolog C
*FOXP4*: Forkhead box P4
*FSIP2*: Fibrous sheath interacting protein 2
*GLI4*: GLI family zinc finger 4
GO: Gene ontology
*GRAMD1C*: GRAM domain containing 1 C
GTF: Gene transfer format
HS: High sensitivity
*HSP90AA1*: Heat shock protein 90 alpha family class A member 1
*ILK*: Integrin linked kinase
*JCHAIN*: Joining chain of multimeric IgA and IgM
*KANSL1L*: KAT8 regulatory NSL complex subunit 1 like
lncRNAs: Long non-coding RNAs
*MAPK6*: Mitogen-activated protein kinase 6
*MED13*: Mediator complex subunit 13
MF: Molecular function
miRNAs: micro RNAs
mRNAs: messenger RNAs
Mt_rRNA: mitochondrial rRNA
MtrRNAs: Mitochondrial ribosomal RNAs
MttRNAs: Mitochondrial transfer RNAs
*NAV3*: Neuron navigator 3
*NCLN*: Nicalin
*NEURL1*: Neuralized E3 ubiquitin protein ligase 1
NS: Non-significant
*OAZ3*: Ornithine decarboxylase antizyme 3
*OS9*: OS9 endoplasmic reticulum lectin
PBS: Phosphate-buffered saline
PCR: Polymerase chain reaction
*PCSK4*: Proprotein convertase subtilisin/kexin type 4
*PGAP2*: Post-GPI attachment to proteins 2
*PHC1*: Polyhomeotic homolog 1
piRNAs: Piwi interacting RNAs
*PRKCD*: Protein kinase C delta
*PRM1*: Protamine 1
*PRM2*: Protamine 2
*RCC2*: Regulator of chromosome condensation 2
*RGS12*: Regulator of G protein signaling 12
RNA-Seq: RNA sequencing
rRNAs: ribosomal RNAs
RT: Room temperature
SAM: Sequence alignment/map
*SDCCAG8*: SHH signaling and ciliogenesis regulator SDCCAG8
*SEC61A2*: Section 61 translocon subunit alpha 2
*SESN1*: Sestrin 1
sncRNAs: short noncoding RNAs
snoRNAs: small nucleolar RNAs
snRNAs: small nuclear RNAs
*SPATA20*: Spermatogenesis associated 20
SRP: Signal recognition particle
*SRP54*: Signal recognition particle 54
*SSFA2*: Sperm-specific antigen 2
*TAX1BP1*: Tax1 binding protein 1
*TBR1*: T-box brain transcription factor 1
TCEP: Tris(2-carboxyethyl)phosphine
*TENT5C*: Terminal nucleotidyltransferase 5 C
*TMEM106B*: Transmembrane protein 106B
*TMEM151A*: Transmembrane protein 151 A
*TMEM260*: Transmembrane protein 260
TNP1: Transition protein 1
TPP2: Tripeptidyl peptidase 2
*TRAM1*: Translocon associated membrane protein 1
tRNAs: transfer RNAs
*TSSK2*: Testis specific serine kinase 2
*TSSK6*: Testis specific serine kinase 6
*UPF2*: UPF2 regulator of nonsense mediated MRNA decay
*USP34*: Ubiquitin specific peptidase 34
*VDAC3*: Voltage dependent anion channel 3
*WASHC1*: WASH complex subunit 1
*WDR13*: WD repeat domain 13
*ZNF644*: Zinc finger protein 644
