# Supplementary material for: Sperm RNA landscape during sexual maturation in Duroc boars
Source: BMC Genomics. 2026 Jan 21;27:197. doi: 10.1186/s12864-025-12490-0 (PMC12906034; doi:10.1186/s12864-025-12490-0)
Supplement: Supplementary file 3 — Supplementary Material 3. [file 12864_2025_12490_MOESM3_ESM.docx]

Table S2: Gene biotype distribution in boar sperm transcriptome. The table shows the relative abundance of different RNA biotypes among 15,637 genes detected by total RNA sequencing after quality filtering (≥10 reads in ≥6 samples).

| Biotype | Count | Percentage |
| --- | --- | --- |
| protein_coding | 13580 | 86.91 |
| lncRNA | 1807 | 11.56 |
| snoRNA | 96 | 0.61 |
| psuedogene | 51 | 0.33 |
| snRNA | 36 | 0.23 |
| scaRNA | 16 | 0.1 |
| miRNA | 8 | 0.05 |
| misc_RNA | 6 | 0.04 |
| processed_pseudogene | 6 | 0.04 |
| unitary_pseudogene | 4 | 0.03 |
| Mt_tRNA | 3 | 0.02 |
| Y_RNA | 3 | 0.02 |
| Mt_rRNA | 2 | 0.01 |
| ribozyme | 2 | 0.01 |
| rRNA | 2 | 0.01 |
| transcribed_unprocessed_pseduogene | 1 | 0.01 |
| translated_unprocessed_pseudogene | 1 | 0.01 |
| vault_RNA | 1 | 0.01 |
