# Supplementary material for: Sperm RNA landscape during sexual maturation in Duroc boars
Source: BMC Genomics. 2026 Jan 21;27:197. doi: 10.1186/s12864-025-12490-0 (PMC12906034; doi:10.1186/s12864-025-12490-0)
Supplement: Supplementary file 9 — Supplementary Material 9. [file 12864_2025_12490_MOESM9_ESM.docx]

# **Additional Files**

Table S1: RNA sequencing quality and alignment metrics for boar sperm samples. Quality metrics are shown for individual boar samples (n = 6 boars; n = 12 samples) across two age groups (~7 months and ~10 months). Metrics include raw read depth, trimming efficiency, mapping rates, and library complexity.

Table S2: Gene biotype distribution in boar sperm transcriptome. The table shows the relative abundance of different RNA biotypes among 15,637 genes detected by total RNA sequencing after quality filtering (≥10 reads in ≥6 samples).

Table S3: Complete list of significantly expressed genes in boar sperm at 10 months versus 7 months age (adj *P* < 0.05) (*n* = 6 boars, 12 samples total). The table includes Ensembl gene ID (ensgene), Ensembl gene symbol (external_gene_name), gene biotype, base mean normalized values (baseMean), log₂ fold change (log_2_FC), standard error of the fold change (lfcSE), Wald test statistics (stat), raw *P*-value, adjusted *P*-value (adj *P*-value), Entrez gene ID, chromosome name, strand, gene description.

Table S4: Complete list of differentially expressed genes in boar sperm at 10 months versus 7 months age (adj *P* < 0.05 & |log_2_FC| ≥ 0.5) (*n* = 6 boars, 12 samples total). The table includes Ensembl gene ID, Ensembl gene symbol, gene biotype, base mean normalized values (baseMean), log₂ fold change (log_2_FC), standard error of the fold change (lfcSE), Wald test statistics (stat), raw *P*-value and adjusted *P*-value (adj *P*-value).

Table S5: Gene Ontology (GO) enrichment analysis of differentially expressed genes in boar sperm at 10 months versus 7 months age (*n* = 6 boars, 12 samples total). The table lists the ontology category (biological process (BP), molecular function (MF)), GO ID, term description, the ratio of target genes versus total targets (GeneRatio), the background ratio (BgRatio), the enrichment factor (RichFactor), fold enrichment, z-score, raw p-value, Benjamini-Hochberg adjusted *P*-value (adj *P*), q-value, NCBI (Entrez) gene IDs, and the count of genes in the term.

Table S6: miRNA sequencing quality metrics for boar sperm samples. Quality metrics are shown for individual boar samples (n = 6 boars; n = 12 samples) across two age groups (~7 months and ~10 months). Metrics include total read counts. Unique reads, duplicate reads, average read length, number of trimmed/filtered reads, retained reads after filtering, overall read assignment rates, mapping rates to mature miRNAs, and mature miRNA read counts.

Table S7: Target genes for differentially expressed miRNAs in boar sperm at 10 months versus 7 months age (*n* = 6 boars, 12 samples total). Tables include miRNAs, the number of target genes (Count) and the Ensembl gene IDs of those targets.

Table S8: Gene ontology (GO) enrichment analysis of target genes for differentially expressed miRNAs in boar sperm at 10 months versus 7 months age (*n* = 6 boars, 12 samples total). The table lists the ontology category , GO ID, term description, the ratio of target genes versus total targets (GeneRatio), the background ratio (BgRatio), the enrichment factor (RichFactor), fold enrichment, z-score, raw p-value, Benjamini-Hochberg adjusted *P*-value (adj *P*), q-value, matching ensembl gene symbols and the count of target genes in the term, and ontology category (biological process (BP), molecular function (MF), cellular component (CC)).
